# *Fusobacterium nucleatum* promotes colorectal cancer through neogenesis of tumor stem cells

**DOI:** 10.1172/JCI181595

**Published:** 2025-02-03

**Authors:** Qinying Wang, Tingting Hu, Qinyuan Zhang, Yichi Zhang, Xiaoxu Dong, Yutao Jin, Jinming Li, Yangyang Guo, Fanying Guo, Ziying Chen, Peijie Zhong, Yongzhi Yang, Yanlei Ma

**Affiliations:** 1 Department of Colorectal Surgery and; 2 Department of Cancer Institute, Fudan University Shanghai Cancer Center (FUSCC), Shanghai, China.; 3 Department of Oncology, Shanghai Medical College, Fudan University, Shanghai, China.

**Keywords:** Gastroenterology, Oncology, Bacterial infections, Cancer, Colorectal cancer

## Abstract

Intestinal stem cells are crucial for maintaining intestinal homeostasis, yet their transformation into tumor stem cells in the context of microbial infection remains poorly understood. *Fusobacterium nucleatum* is frequently associated with the onset and progression of colorectal cancer (CRC). In this study, we uncovered that *F*. *nucleatum* colonized the depths of gut crypts in both patients with CRC and mouse models. Through single-cell sequencing analysis, we demonstrated that *F*. *nucleatum* infection reprogrammed crypt cells and activated lymphocyte antigen 6 complex, locus A^+^ ( LY6A^+^, also known as stem cell antigen 1 [Sca-1]) revival stem cells (RSCs), promoting their hyperproliferation and subsequent transformation into tumor stem cells, which accelerated intestinal carcinogenesis. Mechanistically, we identified LY6A as a glycosylphosphatidylinositol-anchored (GPI-anchored) membrane receptor for *F*. *nucleatum*. Upon binding, *F*. *nucleatum* induced the upregulation of ribosomal protein S14 (RPS14) via the LY6A receptor, driving RSC hyperactivity and tumorigenic conversion. Functional studies showed that genetic ablation of *Ly6a* in intestinal epithelial cells or *Rps14* in LY6A^+^ RSCs substantially reduced *F*. *nucleatum* colonization and tumorigenesis. Moreover, analysis of clinical CRC cohorts revealed a strong correlation between *F*. *nucleatum* infection, RSC expansion, and elevated RPS14 expression in tumor tissues. These findings highlight an alternative *F*. *nucleatum*/LY6A/RPS14 signaling axis as a critical driver of CRC progression and propose potential therapeutic targets for effective CRC intervention.

## Abstract 2

*Fn* induces CRC by initiating tumor stem cells through a LY6A-RPS14 signaling axis.

## Graphical abstract

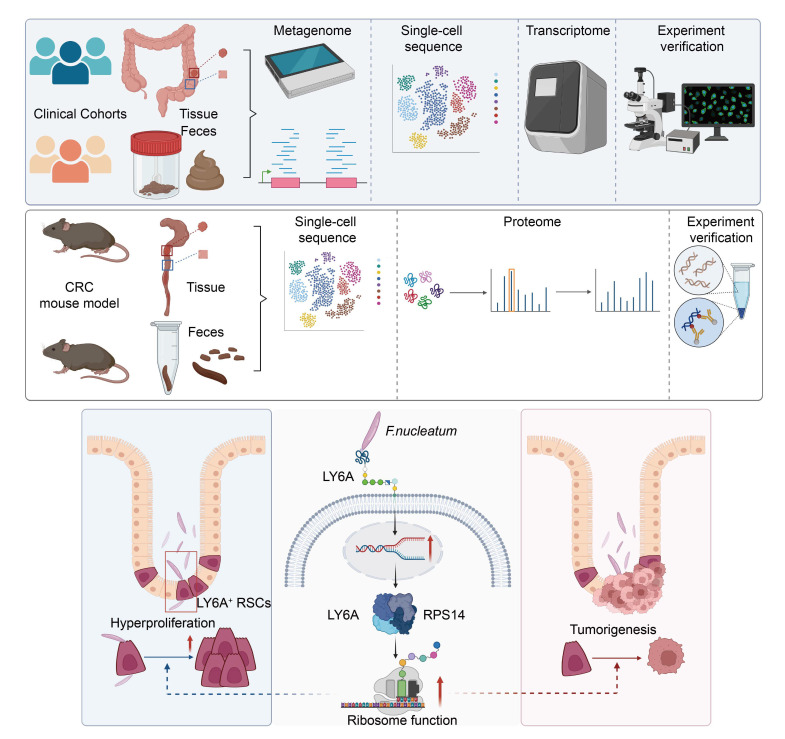

## Introduction

In colorectal cancer (CRC) research, cancer stem cells (CSCs) are recognized as key drivers of tumor initiation, progression, and relapse. Located at the crypt base of the intestine, intestinal CSCs often arise from normal leucine-rich repeat-containing G protein–coupled receptor 5^+^ (LGR5^+^) stem cells and are responsible for epithelial cell renewal (1, 2). Under oncogenic pressures, such as aberrant activation of the Wnt/β-catenin pathway or mutations in key genes including *Apc*, *Kras*, and *Tp53*, the LGR5^+^ stem cells undergo malignant transformation, giving rise to CSCs (3). These CSCs are characterized by their robust self-renewal and pluripotent differentiation capabilities, allowing them to sustain tumor growth, drive metastasis, and resist conventional therapies. Moreover, partially differentiated intestinal progenitor cells can also dedifferentiate into CSCs under specific conditions, such as chronic inflammation, exposure to carcinogens, and pathogenic infections (4). Recently, a novel group of CSCs has been identified, originating from regenerative stem cells or revival stem cells (RSCs), which display a fetal-like transcriptional program (5). These RSCs are particularly responsive to external stimuli and are involved in both tissue regeneration and tumor progression (4, 6, 7). However, the precise mechanisms governing the transition of RSCs from regeneration to tumorigenesis remain poorly understood (8). Lymphocyte antigen 6 complex, locus A (*Ly6a*) (also known as *Sca1*), an 18 kDa glycosylphosphatidylinositol-anchored (GPI-anchored) surface protein of the *Ly6* gene family, has been identified as a marker for RSCs with fetal characteristics. *Ly6a* expression is upregulated during intestinal damage, such as radiation exposure, injury, and inflammation, and is associated with tissue regeneration (9). Additionally, *Ly6a* expression has been reported to increase in response to *Helminth* infections and *Enterococcus faecalis* exposure (10, 11). More recent studies suggest that LY6A^+^ RSCs can function as tumor-initiating stem cells during CRC development (12, 13), with evidence indicating that the activation of fetal-like gene signatures, including Ly6a, during tissue repair may initiate neoplastic transformation if sustained over time (14). These findings raise the possibility that exogenous stimuli, such as pathogenic infections, may disrupt the host’s regenerative homeostasis and contribute to tumor initiation.

With the in-depth exploration of intestinal microecology, increasing attention has been paid to the role of intestinal microbiota in regulating intestinal stem cell activity, notably the involvement of these stem cells in carcinogenesis (15). It has been reported that microbiota may form part of the intestinal stem cell niche and influence both the physiology of normal intestinal stem cells (ISCs) and the pathological behavior of CSCs (16). The bacteria *Helicobacter pylori*, for example, have been shown to interact directly with LGR5^+^ stem cells by promoting their abnormal proliferation, which underscores the role of microbiota as endogenous drivers of tumorigenesis (17). *F*. *nucleatum*, a pathogen frequently detected in CRC tissues, has emerged as a key player in CRC pathogenesis. Clinical correlations between *F*. *nucleatum* infection and CRC include the bacterium’s enrichment in microsatellite instability (MSI) tumors, CpG island methylator phenotype (CIMP) positivity, and right-sided colon tumors (18, 19). *F*. *nucleatum* utilizes various molecular adhesins, including FAP2, FADA, and RADD, to bind to epithelial cells and activate diverse signaling pathways, leading to epithelial hyperplasia and malignant transformation (20–23). While the relationship between *F*. *nucleatum* infection and CRC progression is well established, it remains unclear whether *F*. *nucleatum* directly induces CSCs that initiate and drive tumor progression. Unraveling the interaction between *F*. *nucleatum* and ISCs, along with its effect on the stem cell niche, is essential for understanding the early mechanisms of tumor initiation and identifying potential molecular targets for CRC intervention.

In this study, we combined multiomics approaches with molecular assays to investigate the role of *F*. *nucleatum* in promoting CSC initiation across multiple CRC models, including mouse models, organoids, and patient cohorts. Our findings demonstrate that *F*. *nucleatum* facilitates CSC initiation by interacting directly with the GPI-anchored protein LY6A on the surface of RSCs, leading to the upregulation of ribosomal protein S14 (RPS14), a key ribosomal protein involved in stem cell hyperproliferation. This, in turn, drives the transformation of LY6A^+^ stem cells into tumor-initiating CSCs. Importantly, gut-specific KO of *Ly6a* or ablation of LY6A^+^ stem cells in mouse models abolished the tumorigenic effects of *F*. *nucleatum*, indicating that the interaction of *F*. *nucleatum* with LY6A is essential for CSC initiation. Additionally, human samples and transcriptomics analyses revealed that *F*. *nucleatum* infection upregulated RPS14 expression in tumor tissues, further supporting the role of the *F*. *nucleatum*/RPS14 axis in human CRC. These findings establish the *F*. *nucleatum*/RSC/RPS14 axis as a potential therapeutic target for CRC and underscore the importance of addressing early pathogenic infections in cancer prevention. Future efforts should focus on disrupting the molecular interactions between *F*. *nucleatum* and ISCs to prevent the initiation of CSCs and halt tumor progression at its earliest stages.

## Results

### F. nucleatum colonizes in the gut crypt and activates LY6A^+^ stem cells.

**Figure 1:**
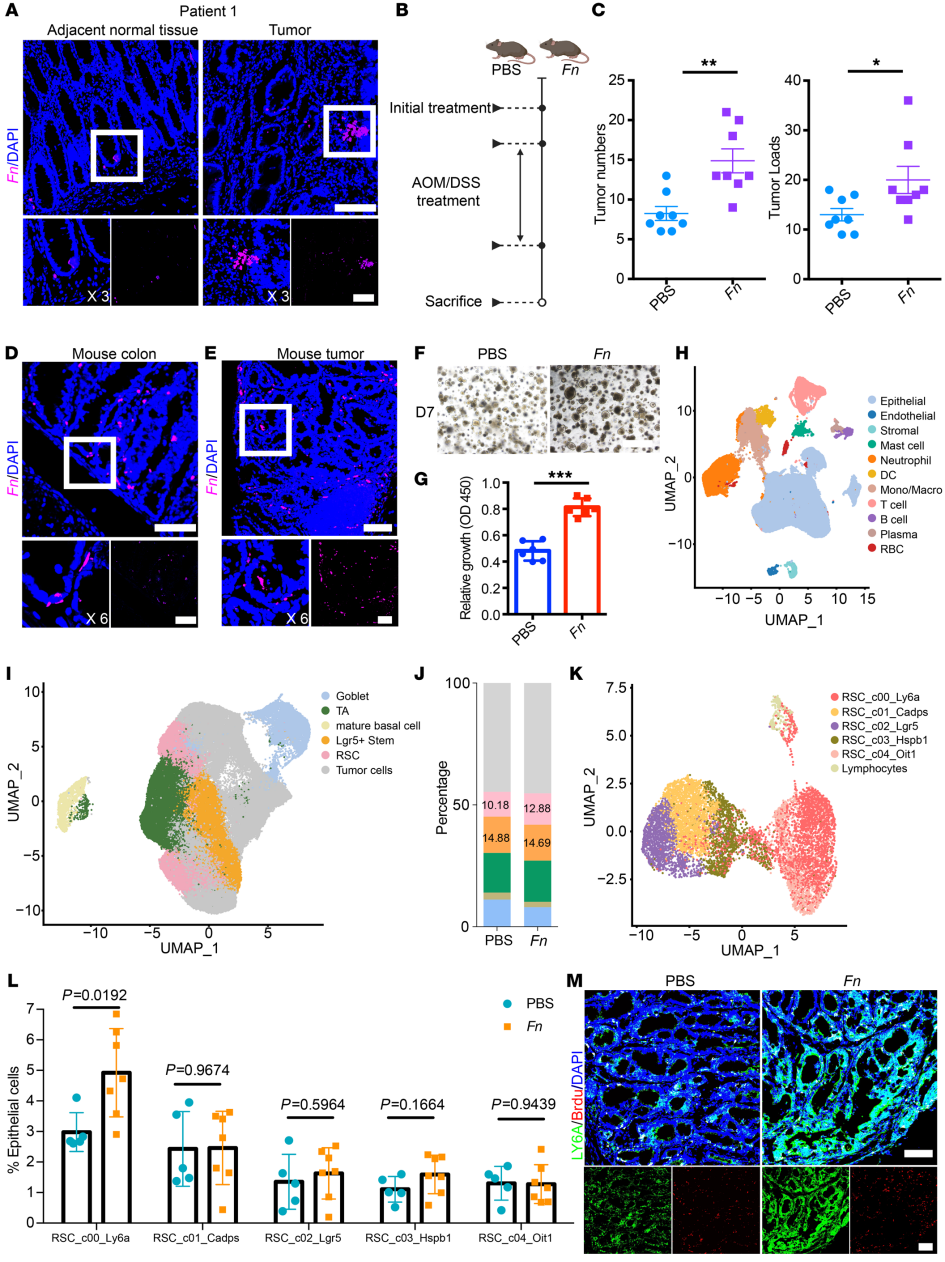
*F*. *nucleatum* colonizes in the gut crypt and increases LY6A^+^ stem cells in tumor tissues. (**A**) FISH assay showing the distribution of *F*. *nucleatum* (*Fn*) (×40, Cy5-conjugated, purple) in the crypts of paracancer and cancer tissues in humans. The white box highlights positive *F*. *nucleatum* staining. (**B**) Schematic of the mouse model protocol. (**C**) Tumor numbers and loads in the entire colon and rectum were measured at the end of the study (*n* = 8). (**D**) FISH assay showing the distribution of *F*. *nucleatum* (×40, Cy5-conjugated, purple) in the crypts of mouse colons. The white box highlights positive *F*. *nucleatum* staining. (**E**) FISH assay showing the distribution of *F*. *nucleatum* (×20, Cy5-conjugated, purple) in mouse tumors. The white arrows indicate positive staining of *F*. *nucleatum*. (**F**) Bright-field images of organoids generated from tumor tissues of mice treated with PBS or *F*. *nucleatum*. (**G**) Growth rate of organoids from tumors from mice treated with PBS or *F*. *nucleatum*. (**H**) Uniform manifold approximation and projection (UMAP) plot showing clusters of total cells in mouse tumors according to scRNA-Seq data (*n* = 5 PBS-treated mice; *n* = 7 *F*. *nucleatum*–treated mice). Mono/Macro, monocyte/macrophage. (**I**) UMAP plot showing clusters of epithelial cells according to scRNA-Seq data. (**J**) Histogram plot of epithelial cell clusters according to scRNA-Seq data. (**K**) UMAP plot showing clusters of RSCs according to scRNA-Seq data. (**L**) Proportion of RSC subgroups in PBS- and *F*. *nucleatum*–treated groups. (**M**) Immunostaining showing the distribution of LY6A and BrdU in tumors from PBS- and *F*. *nucleatum*–treated mice. Scale bars: 100 μm (**A**, **D**, **E**, and **M**) and 1 mm (**F**). Representative data are presented as the mean ± SEM of at least 3 assays. *P < .05, **P < .01, and ***P < .001, by 2-tailed Student’s *t* test (**C** and **G**) and 1-way ANOVA with post hoc Tukey’s multiple-comparison test (**L**).

Given that *F*. *nucleatum* promoted CRC tumorigenesis, we wondered if the cancer-promoting effect related to its temporal progression and spatial distribution. With this question in mind, we collected 19 fresh samples of cancerous and adjacent tissues (cohort 1) to characterize the localization of *F*. *nucleatum* within human gut tissue. Using *F*. *nucleatum* oligonucleotide probes, we performed FISH on colonic tumors and adjacent normal tissues from biopsies, with histological examination of the tumor regions and bacterial infection conducted beforehand (Supplemental Figure 1A; supplemental material available online with this article; https://doi.org/10.1172/JCI181595DS1). Intriguingly, we identified a population of mucus-associated *F*. *nucleatum* microcolonies concentrated within the colonic crypts in 4 cases (Figure 1A and Supplemental Figure 1B). Notably, we observed dysplasia in *F*. *nucleatum*–surrounded crypt cells. Further imaging of human colon sections labeled with an anti–*F*. *nucleatum* antibody revealed a correlation between *F*. *nucleatum* density and dysplasia, demonstrating its interaction with basal cells of the crypt both apically and basally (Supplemental Figure 1C). Given the abundant distribution of stem cells in the crypts, we speculated that *F*. *nucleatum* may directly interact with these stem cells, promoting hyperproliferation and leading to the formation of tumor stem cells.

To validate this hypothesis, we utilized an azoxymethane/dextran sodium sulfate (AOM/DSS) mouse model of spontaneous CRC (Figure 1B). Mice were treated daily with 1 × 10^9^ CFU *F*. *nucleatum* via gavage for over 14 consecutive weeks, while the control group received PBS. Prior to treatment, both groups were dosed with metronidazole for 5 days (Supplemental Figure 2A). *F*. *nucleatum* was detectable in stool samples of mice after gavage (Supplemental Figure 2, B and C). Furthermore, we collected colonic tissues from mice at 1, 2, 3, 7, and 14 days after gavage to assess *F*. *nucleatum* colonization; the results indicated that *F*. *nucleatum* could colonize in intestinal tissue for 1 week after gavage (Supplemental Figure 2D). At the end of the experiment, quantitative PCR (qPCR) revealed that *F*. *nucleatum* primarily colonized submucosal regions within the gut (Supplemental Figure 2E). We evaluated tumor multiplicity and burden and confirmed that *F*. *nucleatum* clearly promoted CRC development and tumor cell proliferation (Supplemental Figure 2, F and G), with *F*. *nucleatum*–treated AOM/DSS mice exhibiting notably higher tumor multiplicities and burdens compared with the PBS-treated group (Figure 1C). FISH and IF assays in mouse gut samples mirrored the results observed in human tissue, demonstrating that *F*. *nucleatum* formed microcolonies in the proliferative zone of crypts, with infection observed in both apical and basal areas (Figure 1D and Supplemental Figure 2H). Additionally, we noted substantial distributions of *F*. *nucleatum* in tumor areas (Figure 1E). These observations, when taken together, shed some light on the effect of *F*. *nucleatum* infection on tumorigenesis from colonization to dysplasia to cancer, a chain reaction of transformations over a long period of time.

Subsequently, tumors from PBS- and *F*. *nucleatum*–treated mice were digested, and cell suspensions were utilized for organoid generation under established protocols. The results showed that the number and area of organoids derived from the *F*. *nucleatum* group far surpassed those from the control PBS group (Figure 1, F and G, and Supplemental Figure 2I), suggesting a direct effect of *F*. *nucleatum* on stem cells. However, the specific groups of stem cells responding to *F*. *nucleatum* infection remain to be identified. To address this, we conducted droplet-based single-cell RNA-Seq (scRNA-Seq) on freshly collected tumor tissue from 5 PBS- and 7 *F*. *nucleatum*–treated mice. After initial quality control and computational integration using the Harmony algorithm, we annotated cell types on the basis of established gene signatures, which yielded 11 major partitions (Figure 1H and Supplemental Figure 3A). The epithelial cells were grouped into categories including goblet cells, transit amplifying cells, mature basal cells, LGR5^+^ stem cells, RSCs, and tumor cells (Figure 1I and Supplemental Figure 3B). Upon comparison of the distributions of cells between the PBS and *F*. *nucleatum* groups, we found that only a subset of RSCs exhibited an increase following *F*. *nucleatum* intervention (Figure 1J). We then isolated RSCs from epithelial cells for further subdivision, distinguishing discrete RSC clusters using gene profiles from the scRNA-Seq datasets (Figure 1K and Supplemental Figure 3C). All 5 subdivisions were shared between the PBS and *F*. *nucleatum* groups (Supplemental Figure 3D), and upon comparison of the RSC population changes across subdivisions, we observed a marked elevation of LY6A^+^ RSCs in the *F*. *nucleatum* group (Figure 1L).

Immunofluorescence (IF) analysis confirmed a denser concentration of LY6A^+^ RSCs within tumors of the *F*. *nucleatum* treatment group compared with the PBS-treated group, alongside a distinct increase in the proliferation of LY6A^+^ cells (Figure 1M). Collectively, these findings demonstrate that *F*. *nucleatum* can establish microcolonies and extensively colonize the crypt region, directly targeting ISCs and promoting an increase in the LY6A^+^ RSC population within tumors. Considering that LY6A^+^ RSCs are known to function as regenerative stem cells under conditions of inflammation and injury, we are left to question how *F*. *nucleatum* infection transforms these regenerative stem cells into tumor stem cells, and what the trajectory of this evolution entails.

### F. nucleatum accelerates colorectal tumorigenesis through promotion of LY6A^+^ RSCs hyperproliferation and transformation.

In order to verify the above query and track the trajectory of how *F*. *nucleatum* regulates the transformation and role of the LY6A^+^ cell population in tumorigenesis, we used *Ly6a^CreERT2^*-knockin mice, in which the Cre recombinase was induced by tamoxifen in LY6A-expressing cells. We assessed the contribution of LY6A^+^ RSCs to colonic carcinogenesis in the context of *F*. *nucleatum* infection (Supplemental Figure 4A). To elucidate the expression pattern of LY6A in the gut, we crossed *Ly6a^CreERT2^* mice with *H11^G/R^* mice, which enabled the expression of ZsGreen and tdTomato through a dual fluorescent reporter system, with loxP sites flanking ZsGreen, integrated into the H11 safe harbor site. This system allows for the distinct expression of green fluorescence (indicating nonrecombination) or red fluorescence (indicating recombination) (Supplemental Figure 4B).

**Figure 2:**
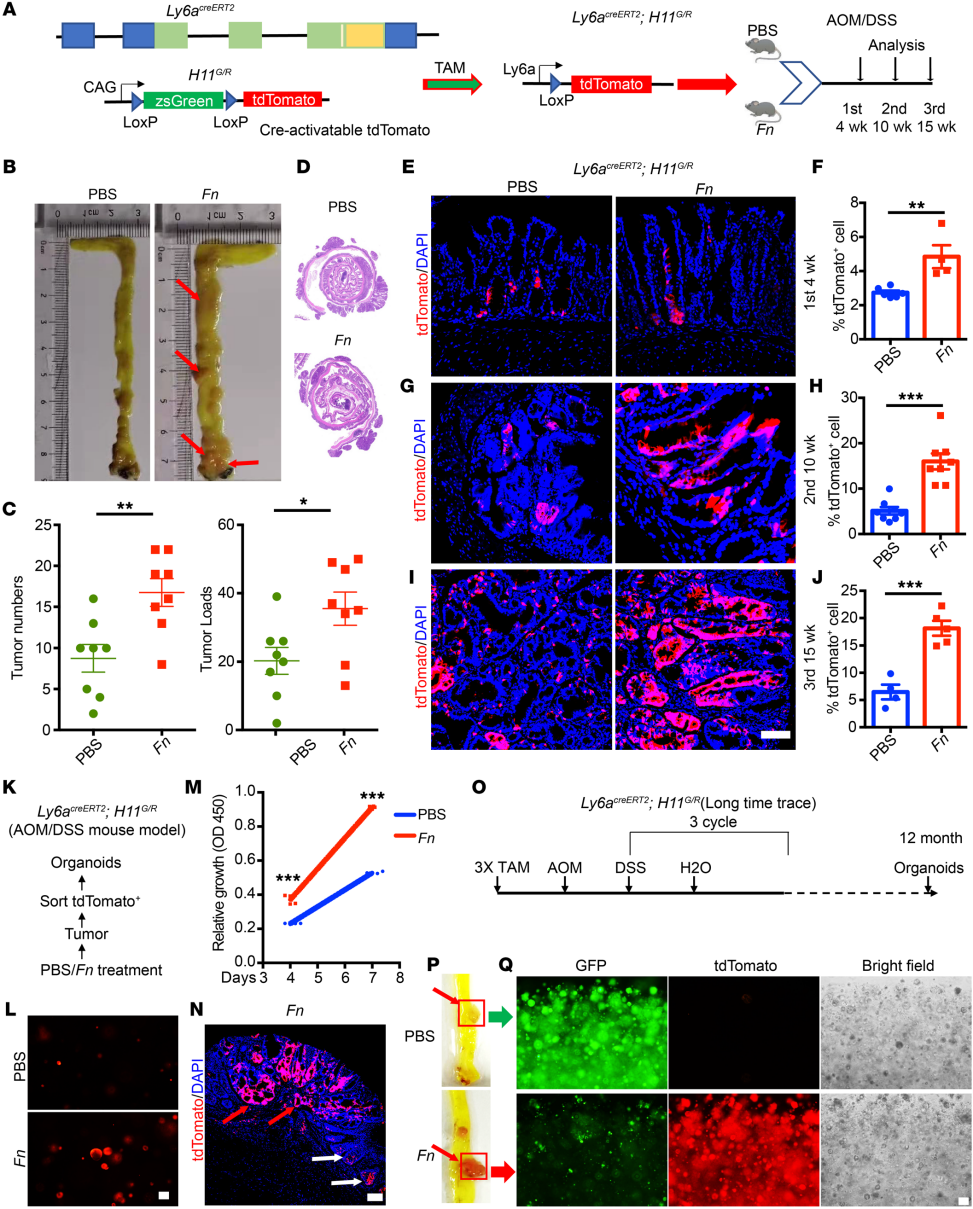
*F*. *nucleatum* can promote the initiation and proliferation of LY6A^+^ tumor stem cells. (**A**) Experimental strategy for tamoxifen-induced, Cre-mediated cell tracking in *Ly6a^CreERT2^ H11^G/R^* mice and a schematic of the experimental design. Arrowheads indicate loxP sites. (**B**) Representative images of the colon and rectum in PBS- and *F*. *nucleatum*–treated groups in the *Ly6a^CreERT2^ H11^G/R^* mouse CRC model, showing tumor numbers and burden. (**C**) Tumor numbers and loads in the colon and rectum measured at the end of the study. (**D**) Representative H&E staining of colons after PBS or *F*. *nucleatum* treatment. (**E**) Representative fluorescence images of lineage-tracing experiments in the colons of *Ly6a^CreERT2^ H11^G/R^* mice treated with PBS or *F*. *nucleatum* at 4 weeks. (**F**) Quantification of LY6A-tdTomato^+^ cells in the colon of *Ly6a^CreERT2^ H11^G/R^* mice treated with PBS or *F*. *nucleatum* at 4 weeks. (**G**) Representative fluorescence images of lineage-tracing experiments in the colons of *Ly6a^CreERT2^ H11^G/R^* mice treated with PBS or *F*. *nucleatum* at 10 weeks. (**H**) Quantification of LY6A-tdTomato^+^ cells in the colons of *Ly6a^CreERT2^ H11^G/R^* mice treated with PBS or *F*. *nucleatum* at 10 weeks. (**I**) Representative fluorescence images of lineage-tracing experiments in the colons of *Ly6a^CreERT2^ H11^G/R^* mice treated with PBS or *F*. *nucleatum* at 15 weeks. (**J**) Quantification of LY6A-tdTomato^+^ cells in the colons of *Ly6a^CreERT2^ H11^G/R^* mice treated with PBS or *F*. *nucleatum* at 15 weeks. (**K**) Schematic of the experimental design showing tamoxifen treatment for LY6A-tdTomato expression and the generation of organoids. (**L**) Representative images of organoids generated by LY6A-tdTomato^+^ cells from tumor tissues after PBS or *F*. *nucleatum* treatment. (**M**) Organoid growth curves for tumors from mice treated with PBS or *F*. *nucleatum*. (**N**) Distribution of LY6A-tdTomato^+^ cells in the inflammatory and inflammatory cancer transformation regions after *F*. *nucleatum* intervention. White arrows indicate regenerative crypts after inflammation, and red arrows indicate areas that transformed into tumors. (**O**) Schematic of the experimental design. (**P**) Tumor development in the PBS- and *F*. *nucleatum*–treated groups after a 12-month long-term tracking period. (**Q**) Organoid generation in the PBS- and *F*. *nucleatum*–treated groups after a 12-month long-term tracking period. Scale bars: 100 μm (**E**, **G**, **I**, **L**, **N**, and **Q**). Representative data are presented as the mean ± SEM of at least 3 assays. *P < .05, **P < .01, and ***P < .001, by 2-tailed Student’s *t* test (**C**, **F**, **H**, **J**, and **M**).

Subsequently, we established the *Ly6a^CreER^* *H11^G/R^* CRC model using the AOM/DSS protocol (Supplemental Figure 4C), alongside non-CRC mice administered PBS or *F*. *nucleatum* via gavage. Sampling points were strategically designed to capture different stages of tumor progression, allowing us to monitor dynamic changes in the LY6A^+^ RSC population during intervention (Figure 2A). At the end of the experiment, we observed an obvious increase in tumor numbers and loads in the *F*. *nucleatum* intervention group compared with the control group (Figure 2, B–D). Notably, a subset of tumors in *F*. *nucleatum*–treated mice exhibited faint red fluorescence, suggesting the presence of LY6A^+^ RSCs as potential tumor stem cells.

We established mouse cohorts with early (4 weeks), middle (10 weeks), and late (15 weeks) infection to monitor the evolution of LY6A-tdTomato^+^ cells during *F*. *nucleatum* infection. Tamoxifen injections commenced 1 week prior to sampling, allowing us to evaluate the distribution and proportions of tdTomato^+^ cells. The results indicated that clusters of LY6A-tdTomato^+^ cells were primarily localized within the crypts of the *F*. *nucleatum* treatment group 4 weeks after infection, showing hyperplastic responses relative to the control group (Figure 2, E and F). As the experiment progressed, we observed substantial hyperplasia in precursor lesions, with a marked upregulation of tdTomato^+^ cells in the *F*. *nucleatum* intervention group (Figure 2, G and H). In the final week, we noted a higher proportion of LY6A-tdTomato^+^ cells within tumors of the *F*. *nucleatum* group, alongside a greater distribution of LY6A-tdTomato^+^ cells in paracancerous tissues compared with controls (Figure 2, I and J, and Supplemental Figure 4D).

To enable quantitative assessment of this phenotype, we performed flow sorting to isolate LY6A-tdTomato^+^ cells from tumor and adjacent tissues from PBS-treated and *F*. *nucleatum*–treated mice for organoid generation (Figure 2K and Supplemental Figure 4E). The results demonstrated that LY6A-tdTomato^+^ stem cells from the *F*. *nucleatum* treatment group formed a greater number of organoids that were larger in volume and had faster growth rates compared with controls (Figure 2, L and M, and Supplemental Figure 4, F and G). In contrast, we observed no differences in the distribution of LY6A-tdTomato^+^ cells between the PBS and *F*. *nucleatum* groups in non-CRC models (Supplemental Figure 4H). Additionally, no discrepancies in organoid volume or proliferation rates were noted (Supplemental Figure 4, I–K). These findings suggest that the effect of *F*. *nucleatum* on the fate of LY6A^+^ stem cells necessitates inflammatory conditions. Inflammation disrupts the intestinal crypt architecture, facilitating the activation of LY6A^+^ RSCs for epithelial repair. Concurrently, inflammation compromises the intestinal barrier, enabling *F*. *nucleatum* accumulation within crypts, which links these 2 events and shifts regenerative processes toward tumor-initiating events driving CRC progression.

To further explore this, we examined specific regions within *F*. *nucleatum*–treated tissues characterized by inflammation and cancer transformation. We found that, although the crypt structures in inflamed regions were compromised, LY6A-tdTomato^+^ RSCs were present at the damaged crypt base. In adjacent tumor areas where inflammation had completed its transformation, we observed a massive accumulation of LY6A-tdTomato^+^ cells with dramatically enhanced vitality (Figure 2N). These observations provide preliminary validation of our hypothesis regarding the capacity of *F*. *nucleatum*–infected, transformed RSCs to act as tumor stem cells. To address the possibility that these transformed stem cells could drive tumor development and contribute to tumor cell differentiation, we designed a long-term tracking experiment (Figure 2O). We administered tamoxifen to mice at the onset of modeling and conducted sampling after 12 months. The results indicated a pronounced red fluorescence in the intestinal tumors of *F*. *nucleatum*–treated mice (Figure 2P). Upon single-cell processing of the red fluorescent tissue to generate organoids, nearly all organoids derived from the *F*. *nucleatum*–treated group were tdTomato^+^, contrasting sharply with those from the PBS-treated group (Figure 2Q). This further substantiates the identity of LY6A^+^ RSCs as tumor-initiating stem cells following *F*. *nucleatum* infection.

Furthermore, we constructed a *Ly6a-DTA* mouse model (Supplemental Figure 5A) by crossing *Ly6a^CreERT2^* mice with Rosa26LSL-DTA mice to create *Ly6a-DTA* mice, in which LY6A^+^ cells were ablated upon tamoxifen treatment (Supplemental Figure 5B). Then, we conducted a PBS and *F*. *nucleatum* intervention with an AOM/DSS model (Supplemental Figure 5C). After tamoxifen dosing, LY6A^+^ stem cells were absent in the normal intestine (Supplemental Figure 5, D and E). We found that LY6A-deficient mice, which are relatively sensitive to AOM and DSS treatment, underwent continuous weight loss, with some of them dying after treatment with these reagents. Despite this, at the end of the experiment, the results showed that *F*. *nucleatum*’s promotion of tumor occurrence and development was reduced after specific ablation of the LY6A^+^ stem cells (Supplemental Figure 5, F and G). Collectively, these results underscore the ability of *F*. *nucleatum* to transform LY6A-tdTomato^+^ RSCs into tumor-initiating stem cells, thereby facilitating the initiation and progression of CRC. However, the underlying mechanisms governing the selective targeting LY6A^+^ cells by *F*. *nucleatum* remain to be elucidated.

### F. nucleatum promotes intestinal tumorigenesis in a LY6A-dependent manner and increases colonic LY6A expression to enhance attachment and gut colonization.

**Figure 3:**
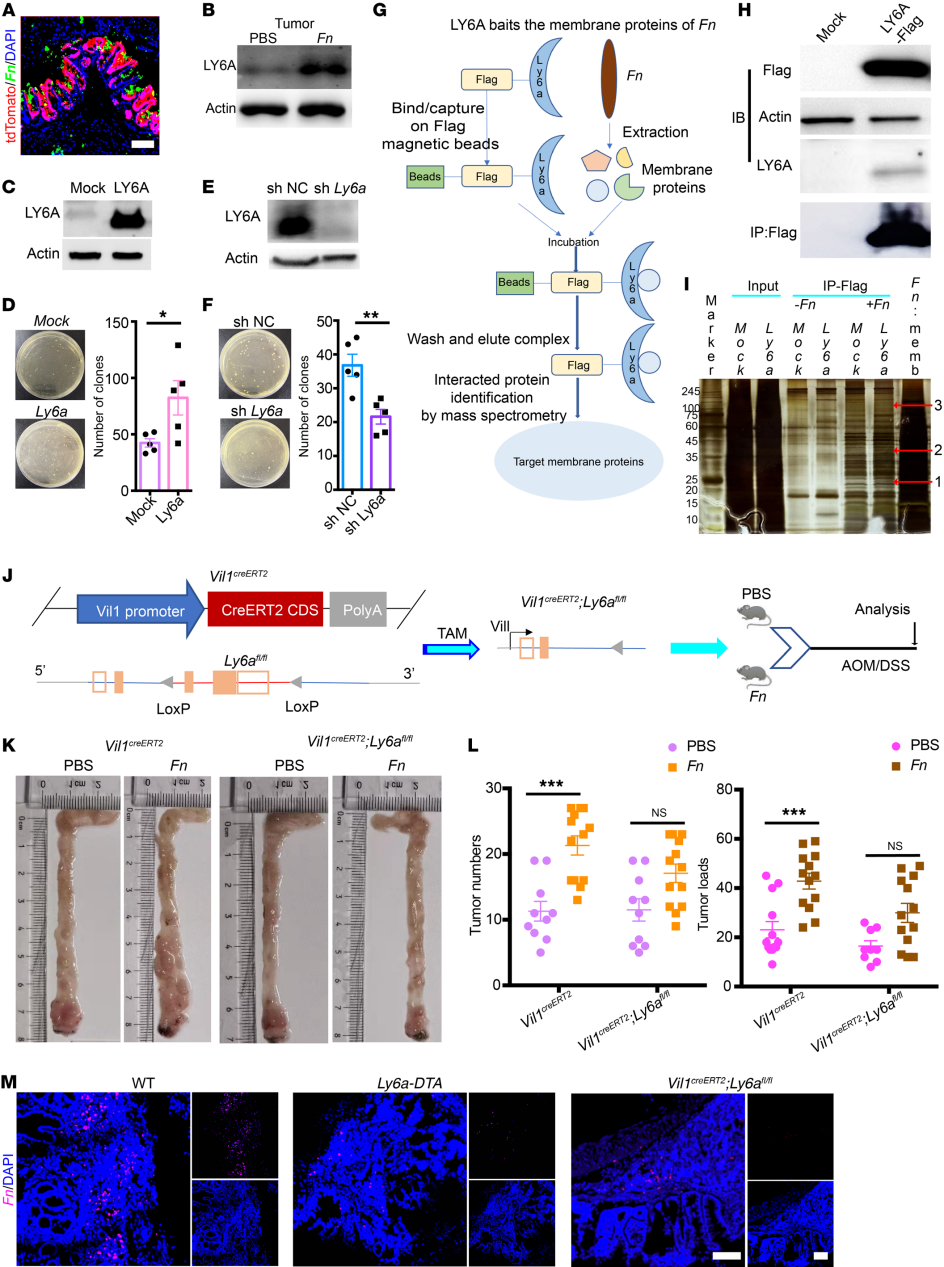
LY6A-specific interaction with *F*. *nucleatum* surface membrane proteins. (**A**) FISH assay detecting *F*. *nucleatum* in direct contact with LY6A-tdTomato^+^ cells in mouse colon. (**B**) Representative Western blots of LY6A expression in tumor tissues from PBS- and *F*. *nucleatum*–treated mice. (**C**) Exogenous LY6A expression in HEK293 cells detected by immunoblotting. (**D**) Exogenous LY6A expression in HEK293 cells increased *F*. *nucleatum* attachment, as shown by adhesion assay. (**E**) Knockdown of LY6A in CT26 cells detected by immunoblotting. shNC, negative control shRNA. (**F**) Knockdown of LY6A reduced *F*. *nucleatum* attachment in CT26 cells, as shown by adhesion assay. (**G**) Schematic diagram showing *F*. *nucleatum*–specific surface membrane proteins interacting with LY6A, identified by IP–mass spectrometry. (**H**) Exogenous Flag-LY6A expression detected by immunoblotting. (**I**) Screening of *F*. *nucleatum* adhesins by IP assay and silver staining. (**J**) Strategy for specific *Ly6a* conditional KO in the gut. (**K**) Representative images of tumor burden in the colon and rectum of PBS- and *F*. *nucleatum*–treated *Vil1^CreERT2^ Ly6a^fl/fl^* mice. (**L**) Tumor numbers and loads in the colon and rectum, measured at the end of the study. (**M**) *F*. *nucleatum* distribution within tumors of WT, *Vil1^CreERT2^ Ly6a^fl/fl^*, and *Ly6a-DTA* mice. Scale bars: 100 μm (**A** and **M**). Representative data are presented as the mean ± SEM of at least 3 assays. *P < .05, **P < .01, and ***P < .001, by 2-tailed Student’s *t* test (**D** and **F**) and 2-way ANOVA with post hoc Tukey’s multiple-comparison test (**L**).

We hypothesized that *F*. *nucleatum* preferentially attached to LY6A^+^ stem cells and speculated whether LY6A, a GPI-anchored surface protein, serves as a specific binding target for *F*. *nucleatum*. To explore this, we used the *Ly6a^CreER2^ H11^G/R^* mouse model and found that *F*. *nucleatum* was closely associated with LY6A-tdTomato^+^ cells, from the gut entrance to the colonic crypts (Figure 3A and Supplemental Figure 6, A and B). Interestingly, *F*. *nucleatum* treatment not only expanded the proportion of LY6A^+^ cells but also upregulated LY6A expression in tumor tissues (Figure 3B). These data suggest that *F*. *nucleatum* may exert its oncogenic effect through direct interaction with LY6A.

Next, we evaluated the adhesion properties of *F*. *nucleatum* in vitro using HEK293 and CT26 cell lines. The bacterial adhesion assay revealed that *F*. *nucleatum* exhibited higher attachment to LY6A-overexpressing cells compared with mock controls (Figure 3, C and D). Consistent with this, knockdown of *Ly6a* in CT26 cells using siRNA markedly reduced *F*. *nucleatum* attachment (Figure 3, E and F), further supporting the role of LY6A in facilitating *F*. *nucleatum* colonization.

To identify the specific proteins mediating *F*. *nucleatum* attachment to LY6A, we induced overexpression of Flag-tagged LY6A and performed IP using anti-Flag magnetic beads, followed by incubation with membrane proteins isolated from *F*. *nucleatum*. The SDS-PAGE bands were excised and analyzed by mass spectrometry, identifying nearly 100 *F*. *nucleatum* proteins potentially interacting with LY6A (Figure 3, G–I, and Supplemental Table 1). Collectively, these findings indicate that *F*. *nucleatum* promotes the transformation of LY6A^+^ RSCs into tumor stem cells by binding specifically to LY6A, thus driving colorectal tumorigenesis.

### Decreased F. nucleatum distribution in the mouse crypts and tumors after LY6A deletion or LY6A^+^ cell ablation.

To further confirm whether the pro-CRC effect of *F*. *nucleatum* was LY6A dependent in vivo, we generated gut-specific *Ly6a*-KO mice by crossing *Vil1^CreERT2^* mice with *Ly6a*-floxed (*Ly6a^fl/fl^*) mice, resulting in *Villin^CreER2^* *Ly6a^fl/fl^* offspring (Figure 3J and Supplemental Figure 6, C and D). These mice were viable and exhibited no gross phenotypic abnormalities compared with their WT littermates.

To assess the role of LY6A in *F*. *nucleatum*–driven tumorigenesis, *Villin^CreER2^* *Ly6a^fl/fl^* mice and their WT counterparts received daily oral administration of either PBS or *F*. *nucleatum* (Supplemental Figure 6E). This model allowed conditional deletion of *Ly6a* specifically in the gut. *Villin^CreER2^* *Ly6a^fl/fl^* mice displayed efficient *Ly6a* deletion in both small and large intestines, as confirmed by tissue analysis (Supplemental Figure 6, F and I). The results showed that *Ly6a* deletion led to a noted reduction in both tumor number and tumor load in the *F*. *nucleatum*–treated *Villin^CreER2^* *Ly6a^fl/fl^* mice compared with WT controls (Figure 3, K and L). Additionally, the distribution of *F*. *nucleatum* within colonic tumors and crypts was markedly reduced in *Villin^CreER2^* *Ly6a^fl/fl^* mice and *Ly6a-DTA* mice (Figure 3M and Supplemental Figure 6J). These findings strongly suggest that *F*. *nucleatum* directly targeted LY6A^+^ RSCs to promote their transformation into tumor-initiating cells, thereby mediating colorectal tumorigenesis. However, the precise molecular mechanisms underlying this interaction remain unclear.

### F. nucleatum triggers LY6A^+^ tumor stem cells through upregulation of RPS14 expression.

**Figure 4:**
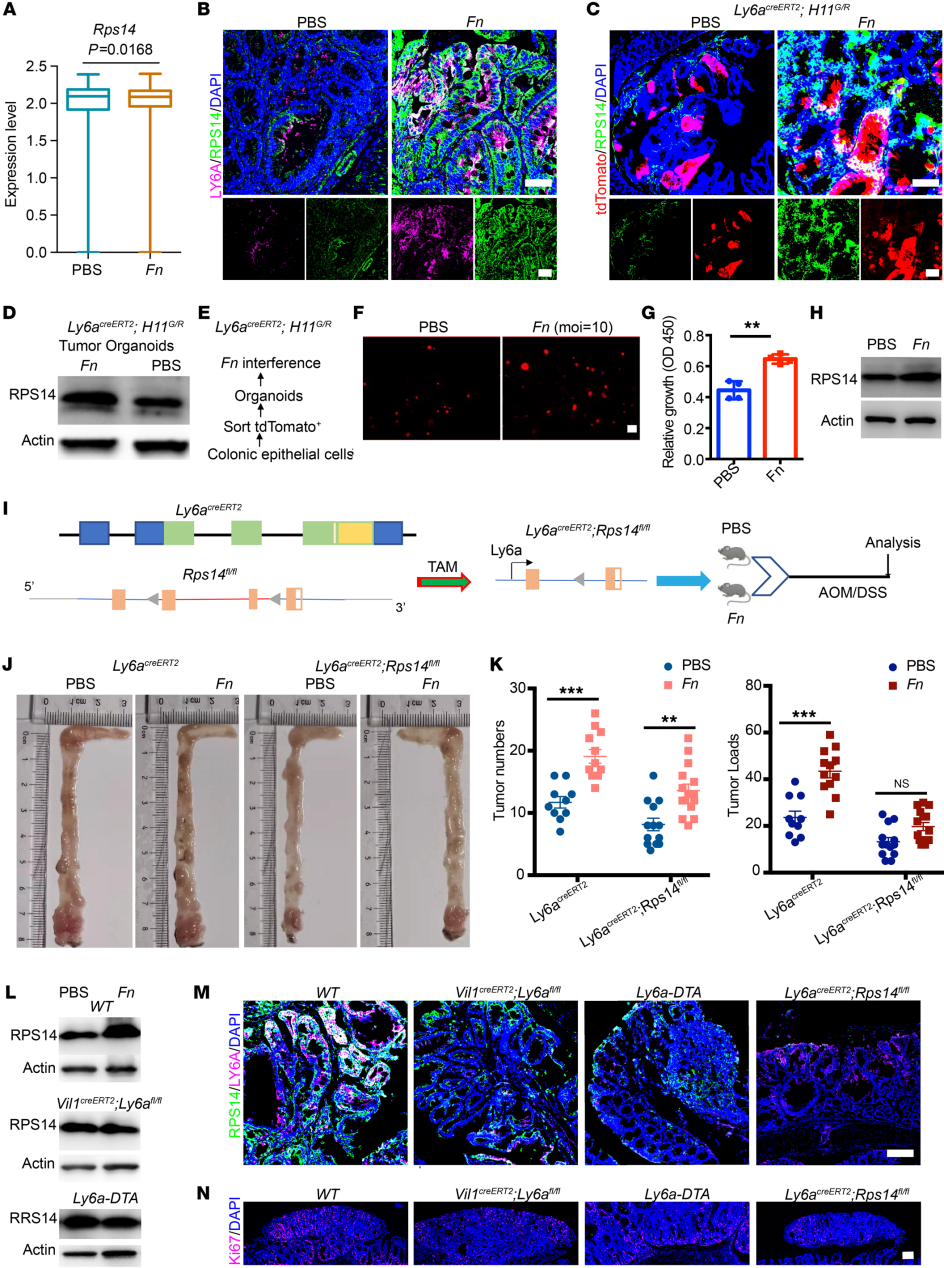
*F*. *nucleatum* induces RPS14 upregulation in LY6A^+^ cells. (**A**) Box plot showing that *Rps14* was specifically upregulated in LY6A^+^ RSCs after *F*. *nucleatum* intervention. (**B**) Representative IF costaining for LY6A and RPS14 in tumors from PBS- and *F*. *nucleatum*–treated mice. (**C**) RPS14 expression in LY6A-tdTomato^+^ RSCs in *Ly6a^CreERT2^ H11^G/R^* mice treated with PBS or *F*. *nucleatum*. (**D**) RPS14 expression in LY6A-tdTomato^+^ RSC–derived organoids from tumors treated with PBS or *F*. *nucleatum*. (**E**) Schematic of the experimental design showing tamoxifen treatment to induce LY6A-tdTomato expression and organoid generation. (**F**) Representative image of organoids generated from LY6A-tdTomato^+^ RSCs in normal epithelial tissue and treated with PBS or *F*. *nucleatum*. (**G**) Organoid growth rate after PBS or *F*. *nucleatum* intervention. (**H**) RPS14 expression after PBS or *F*. *nucleatum* intervention. (**I**) Experimental procedure to target *Rps14* in LY6A^+^ cells. (**J**) Representative images of the colon and rectum in the PBS- and *F*. *nucleatum*–treated *Ly6a^CreERT2^ Rps14^fl/fl^* CRC mouse model, showing tumor burden. (**K**) Tumor numbers and loads in the colon and rectum measured at the end of the study. (**L**) Immunoblot showing that *F*. *nucleatum*–mediated upregulation of RPS14 expression was blocked in WT, *Vil1^CreERT2^ Ly6a^fl/fl^*, and *Ly6a-DTA* mice treated with *F*. *nucleatum*. (**M**) Representative images of LY6A and RPS14 staining in tumors of WT, *Vil1^CreERT2^ Ly6a^fl/fl^*, *Ly6a-DTA*, and *Ly6a^CreERT2^ Rps14^fl/fl^* mice treated with *F*. *nucleatum*. (**N**) Representative images of Ki67 staining in tumors from WT, *Vil1^CreERT2^ Ly6a^fl/fl^*, *Ly6a-DTA*, and *Ly6a^CreERT2^ Rps14^fl/fl^* mice treated with *F*. *nucleatum*. Scale bars: 100 μm (**B**, **C**, **F**, **M**, and **N**). The data are presented as the mean ± SEM of at least 3 assays. **P < .01 and ***P < .001, by 2-tailed Student’s *t* test (**A** and **G**) and 2-way ANOVA with post hoc Tukey’s multiple-comparison test (**K**).

To elucidate the molecular link between *F*. *nucleatum* and LY6A^+^ stem cell behavior, we first conducted single-cell transcriptomics analysis to identify the genes upregulated in LY6A^+^ RSCs following *F*. *nucleatum* exposure. Our results revealed a marked upregulation of *RPS14* (Figure 4A), a component of the 40S ribosomal subunit that plays a pivotal role in ribosome biogenesis and function (24). Notably, RPS14 expression was obviously elevated in LY6A^+^ RSCs derived from *F*. *nucleatum*–treated tumor tissues (Figure 4B). Further investigation using *F*. *nucleatum*–exposed *Ly6a^CreERT2^ H11^G/R^* mouse models revealed a similar upregulation of RPS14 expression in LY6A^+^ RSCs within tumor tissues (Figure 4C). In parallel, organoids generated from LY6A-tdTomato^+^ RSCs, isolated from *F*. *nucleatum*–treated tumors, also exhibited a visible increase in RPS14 expression (Figure 4D).

For direct validation of these findings, we performed in vitro experiments by first administering tamoxifen to *Ly6a^CreERT2^ H11^G/R^* mice, followed by isolation of Ly6a-tdTomato^+^ cells to generate organoids and then treatment of these organoids with either PBS or *F*. *nucleatum* (Figure 4E). The results showed a pronounced increase in both the organoid size and proliferation rate following *F*. *nucleatum* treatment, accompanied by apparent upregulation of RPS14 expression (Figure 4, F–H). These observations suggest that RPS14 upregulation was linked to the hyperproliferation of LY6A^+^ stem cells and their transformation into tumor stem cells. In support of this observation, IF analysis revealed obvious enrichment of KI67, a marker of proliferation, in regions with high RPS14 expression (Supplemental Figure 7A).

To further confirm that *F*. *nucleatum* mediates tumor stem cell initiation and progression through RPS14 upregulation in LY6A^+^ RSCs, we generated *Ly6a^CreER^ RPS14^fl/fl^* mice to achieve conditional KO of RPS14 in LY6A^+^ cells (Figure 4I and Supplemental Figure 7B). Using this model, we established an *F*. *nucleatum*–treated AOM/DSS CRC mouse model (Supplemental Figure 7C). We observed efficient ablation of RPS14 in LY6A^+^ stem cells in *LY6A^CreERT2^ RPS14^fl/fl^* mice following tamoxifen injection (Supplemental Figure 7D).

Our results further demonstrated that specific RPS14 ablation in LY6A^+^ stem cells greatly attenuated the tumor-promoting effects of *F*. *nucleatum*, resulting in a substantial reduction in tumor burden within the colon (Figure 4, J and K). However, we also observed that the increase in tumor number induced by *F*. *nucleatum* was not completely abolished after RPS14 KO in LY6A^+^ cells. Considering the correlation between high RPS14 expression and tumor stem cell proliferation, we speculated that KO of RPS14 may inhibit tumor growth by halting progression at the small adenoma stage. Subsequent analysis confirmed this observation when tumors larger than 200 μm were categorized for statistical analysis. The results showed that, in the group in which RPS14 was specifically knocked out in LY6A^+^ stem cells, the number of tumors in the *F*. *nucleatum*–treated group was significantly lower than that in the *F*. *nucleatum*–treated WT group (Supplemental Figure 7E). This suggests that RPS14 played a key role in facilitating the tumor-promoting effects of *F*. *nucleatum*.

To further substantiate these findings, we crossed *Ly6a^CreERT2^ H11^G/R^* mice with *Ly6a^CreER^ RPS14^fl/fl^* mice to generate *Ly6a^CreET2R^ H11^G/R^ RPS14 ^fl/fl^* mice (Supplemental Figure 7F). Following tamoxifen injection, we successfully isolated LY6A-tdTomato^+^ stem cells and generated organoids (Supplemental Figure 7, G and H). Upon RPS14 KO in these LY6A-tdTomato^+^ RSCs, we observed an increased propensity for differentiation, and the *F*. *nucleatum*–induced proliferative phenotype in the organoids was completely abolished (Supplemental Figure 7, L and M). Additional experiments in several mouse models demonstrated that specific *Ly6a* KO in the intestine, LY6A^+^ cell ablation, or *Rps14* KO in LY6A^+^ cells led to a noted reduction in RPS14 expression and tumor cell proliferation (Figure 4, L–N). Collectively, these results strongly support our hypothesis that *F*. *nucleatum* drives CRC progression via RPS14 upregulation in LY6A^+^ RSCs.

To gain deeper insights into the physiological changes associated with RPS14 upregulation, we performed proteomics analysis of tumor tissues from PBS- and *F*. *nucleatum*–treated WT, *Villin^CreER2^* *Ly6a^fl/fl^*, and *Ly6a^CreER^ RPS14^fl/fl^* mice. The proteomics profiles revealed substantial differences in protein expression across the groups (Supplemental Figure 8A). Notably, the proteomics data highlighted that *F*. *nucleatum*–treated mice exhibited altered biological processes, including ribosomal function and pathogen-related pathways (Supplemental Figure 8B). Ribosomes, particularly heterogeneous ones in stem cells, display variable rRNA features and ribosomal protein compositions, allowing for selective mRNA translation and regulation of protein abundance in response to external stimuli (25). Our data suggest that *F*. *nucleatum* infection modulated ribosomal function, leading to these observed biological changes. Importantly, *L y6a* or *Rps14* KO clearly attenuated these *F*. *nucleatum*–mediated processes, confirming the critical role of RPS14 in the tumor-promoting effects of *F*. *nucleatum* (Supplemental Figure 8, C and D).

### F. nucleatum infection promotes activation of RSCs and high expression of RPS14 in patients with CRC.

**Figure 5:**
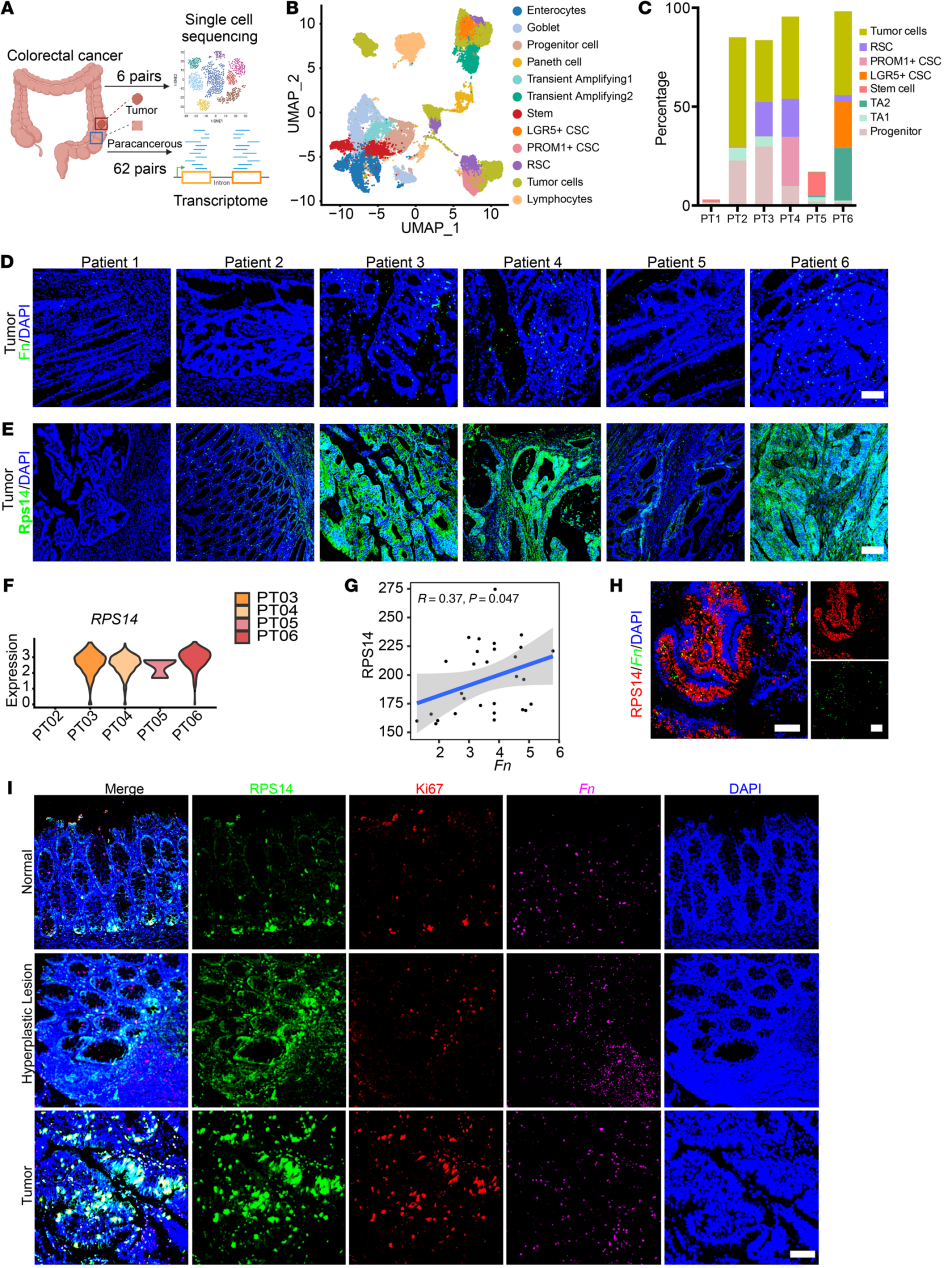
*F*. *nucleatum* infection induces the activation of tumor RSCs and high expression of RPS14 in patients with CRC. (**A**) Schematic of the experimental design. Created with BioRender.com. (**B**) UMAP plot showing epithelial clusters in scRNA-Seq data (PT1-PT6). (**C**) Distribution statistics of cell proportions in each sample group (patients 1–6 [PT1–PT6]). (**D**) IF assay detecting *F*. *nucleatum* distribution in human cancer tissues (PT1–PT6). (**E**) Representative IF images of RPS14 in human CRC tumor tissue (PT1–PT6). (**F**) Violin plots showing *RPS14* expression levels in RSCs. (**G**) Correlation between high *F*. *nucleatum* infection and *RPS14* expression in patients. (**H**) IF of RPS14 and *F*. *nucleatum* in a representative human CRC tumor section. (**I**) IF images of RPS14, KI67, and *F*. *nucleatum* in normal and tumor tissue sections, showing a positive correlation between RPS14, Ki67 expression, and *F*. *nucleatum* infection. Scale bars: 100 μm (**D**, **E**, **H**, and **I**).

Building on these findings, we sought to investigate whether the *F*. *nucleatum*/RSC/RPS14 axis followed a similar pattern in the context of human CRC development. To this end, we collected a total of 136 tissue samples (cohorts 1 and 4), consisting of cancerous and adjacent normal tissues from 68 patients with CRC. Transcriptome sequencing was performed on 62 tumor and matched adjacent tissues (cohort 2), while single-cell transcriptome sequencing was carried out on 6 matched pairs of tumor and adjacent tissues (cohort 3) (Figure 5A). After applying stringent quality control and computational integration using the Harmony algorithm, we obtained high-resolution single-cell sequencing data. These cells were classified into 11 major lineages on the basis of characteristic phenotypic markers (Supplemental Figure 9A). Within the epithelial compartment, we further identified subpopulations including goblet cells, transit-amplifying cells, mature basal cells, LGR5^+^ stem cells, RSCs, and tumor cells based on established marker profiles (Figure 5B). Notably, the cell heterogeneity was higher in human colonic tissues, with RSCs being exclusively localized within the tumor (Figure 5C).

We assessed the spatial distribution of *F*. *nucleatum* by FISH. Interestingly, we found that *F*. *nucleatum* infection was absent in tumor samples from patients 1 and 2, whereas patient 5 exhibited minimal infection. In contrast, patients 3, 4, and 6 had extensive *F*. *nucleatum* colonization in tumor tissue, while *F*. *nucleatum* infection in adjacent normal tissue was low and dispersed, but a few crypt distributions were visible (Figure 5D and Supplemental Figure 9E). Strikingly, the presence of RSCs closely mirrored *F*. *nucleatum* infection patterns. Patients with high levels of *F*. *nucleatum* infection (patients 3, 4, and 6) also exhibited a marked enrichment of RSCs within tumor tissues, whereas patients with minimal or absent *F*. *nucleatum* infection (patients 1, 2, and 5) showed correspondingly lower RSC distribution (Supplemental Figure 9, B–D). These data strongly suggest that *F*. *nucleatum* may play a critical role in mobilizing RSCs.

Further supporting this, RPS14 expression displayed a concordant trend with the distribution of RSCs, showing higher expression in *F*. *nucleatum*–infected regions (Figure 5, E and F). To delve deeper into this association, we analyzed the transcriptomic data from 62 patients with CRC, focusing on the relationship between *F*. *nucleatum* infection and *RPS14* expression. Patients with elevated *F*. *nucleatum* infection, determined via metagenomic analysis of stool samples, had a prominent positive correlation between *F*. *nucleatum* infection levels and *RPS14* expression within tumor tissues (Figure 5G). Colocalization assays using FISH and IF further confirmed the elevated expression of RPS14 in *F*. *nucleatum*–enriched regions (Figure 5H).

Moreover, through histological analysis of *F*. *nucleatum*–infected cancerous and adjacent tissues (cohort 1), we observed an upregulation of RPS14 expression concomitant with increased cellular proliferation, specifically within *F*. *nucleatum*–enriched regions. These regions included noncancerous adjacent normal tissue (precancerous), paracancerous adenomas (early-stage adenoma), and fully developed cancerous lesions (Figure 5I). This evidence suggests the emergence of a complete timeline in human samples, tracing the progression from *F*. *nucleatum* infection to RPS14 upregulation and subsequent tumor development. It also implies that the high expression of RPS14 and mobilization of RSCs at the early stage of *F*. *nucleatum* infection may have laid the foundation for the subsequent generation of tumor stem cells and the occurrence and development of CRC.

**Figure 6:**
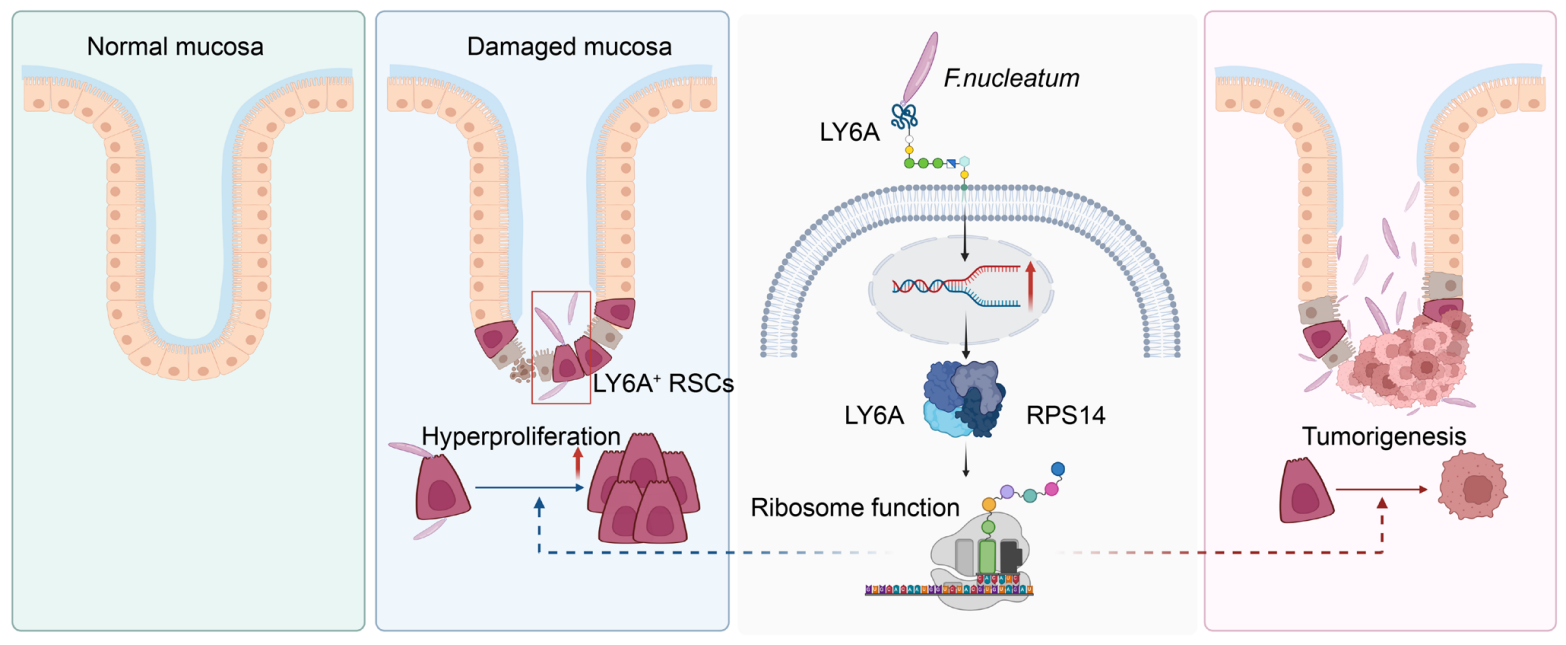
Schematic diagram of the mechanism. (**A**) Under inflammatory conditions, damage to the intestinal barrier activates LY6A^+^ RSCs, initiating the repair process. However, when this damage is accompanied by *F*. *nucleatum* infection, a portion of *F*. *nucleatum* crosses the compromised intestinal barrier, translocating and accumulating at the base of the colonic crypts. There, it directly interacts with LY6A^+^ RSCs through LY6A. This interaction with LY6A protein regulates the downstream overexpression of RPS14, leading to hyperproliferation. As a result, *F*. *nucleatum* hijacks these RSCs, originally intended for tissue regeneration, and converts them into tumor-initiating stem cells, driving the progression of CRC. Figure was created with BioRender.com.

Collectively, these findings reveal a underlying mechanism upon *F*. *nucleatum* in CRC progression. Under inflammatory conditions, damage to the intestinal barrier activates LY6A^+^ RSCs, initiating the repair process. However, when this damage is accompanied by *F*. *nucleatum* infection, a portion of *F*. *nucleatum* crosses the compromised intestinal barrier, translocating and accumulating at the base of the intestinal crypts. There, it directly interacts with LY6A^+^ RSCs through surface molecules. This interaction with LY6A protein regulates the downstream overexpression of RPS14, leading to hyperproliferation. As a result, *F*. *nucleatum* hijacks these RSCs, originally intended for tissue regeneration, and converts them into tumor-initiating stem cells, driving the progression of CRC (Figure 6). These insights not only delineate the pivotal role of *F*. *nucleatum* in CRC progression but also highlight RPS14 as a potential therapeutic target for preventing *F*. *nucleatum*–associated CRC development.

## Discussion

Chronic inflammation is recognized as a key driver of tumorigenesis, with disruption of the intestinal epithelial barrier and the translocation of gut microbiota or their antigens acting as critical initiators. This process often engages the body’s intrinsic resistance mechanisms, including immune responses and the activation of RSCs to facilitate mucosal repair. However, under certain conditions, errors in the damage repair process can give rise to tumor initiation. Therefore, identifying the mediators that convert mucosal healing errors into tumorigenic events is crucial for understanding inflammation-driven carcinogenesis.

CSCs, which drive tumor initiation and progression, can originate from both LGR5^+^ stem cells and LGR5^–^ RSCs (e.g., those expressing clusterin (CLU) and LY6A). These stem cell populations are regulated by a complex interplay of genetic and environmental factors, including the microbiota, that has been shown to influence stem cell behavior, intestinal homeostasis, and tumor development (26–28). Our study utilized scRNA-Seq to examine the effects of *F*. *nucleatum* on LY6A^+^ stem cells in a mouse model of CRC. We observed that *F*. *nucleatum* infection led to an expansion of the LY6A^+^ stem cell population, consistent with their transformation into tumor-initiating cells. This transformation was further confirmed by lineage-tracing studies using LY6A reporter mice, which allowed us to track the behavior of LY6A^+^ stem cells from a healthy state to dysplasia and tumor formation. At 3 distinct time points in our CRC mouse model, we observed that 1 month after *F*. *nucleatum* intervention, LY6A-tdTomato^+^ stem cells localized to the crypt base had formed small clusters with a dysplastic tendency. In the mid-stage of infection, large clusters of LY6A-tdTomato^+^ cells were observed in dysplastic areas. In the late stage of tumorigenesis, LY6A-tdTomato^+^ tumor stem cells remained concentrated in the tumor core. These observations suggest that *F*. *nucleatum* drove the early evolution of LY6A^+^ stem cells into tumor stem cells, with these cells playing a more prominent role in tumor initiation.

Further investigation into the mechanism underlying this transformation revealed that *F*. *nucleatum* colonized crypts, interacted directly with LY6A^+^ stem cells, and altered their cellular properties, leading to abnormal hyperproliferation and tumor initiation. LY6A^+^ cells, characterized by their fetal transcriptional profile, emerge in response to injury and are involved in epithelial regeneration (9). Our study provides a mechanistic explanation for the complex interplay between regeneration and tumorigenesis after injury and infection by pathogenic microorganisms. *F*. *nucleatum* infection appears to prolong the normally short-lived regenerative response of LY6A^+^ RSCs, promoting neoplasia.

We observed that in human CRC tissue samples, *F*. *nucleatum* infection was associated with the presence of RSCs. The sequence of human *LY6A* orthologous genes is being studied and has not yet been established for single-cell sequencing analysis. We found that CCND2, a molecular marker of RSCs, was positively correlated with *F*. *nucleatum* infection in transcriptomic data from cohort 4 (data not shown). Comparative analysis of our scRNA-Seq data with data from Ayyaz et al. (7) revealed that LY6A^+^ cells were enriched in the subpopulations SSC2a and SSC2c, with SSC2c expressing high levels of CCND2. This suggests that the LY6A^+^ cell population may include a subset of CCND2^+^ RSCs and provides further insights for future research into the role of RSCs in tumorigenesis. Additionally, our findings demonstrate the translational relevance of the mouse model in recapitulating *F*. *nucleatum*–induced tumor stem cell generation and CRC progression in humans.

Clinically, *F*. *nucleatum* is more prevalent in the right-sided proximal colon, where it is associated with fetal-like progenitor phenotypes and increased *Ly6a* expression (12, 29). Similar upregulation of *Ly6a* has been observed in response to helminth infection (30). In our experiments, we found that *F*. *nucleatum* infection upregulated LY6A expression in intestinal tissues. However, this upregulation did not correspond to an overall increase in stem cell populations, leading us to consider several factors: the role of LY6A in tumorigenesis, its function in tissue repair, and its potential as a pathogen receptor. *F*. *nucleatum* surface proteins specifically recognize and bind to LY6A, facilitating the colonization of LY6A^+^ stem cells and driving their transformation into tumor stem cells. Specific knockdown of *Ly6a* and ablation of LY6A^+^ cell populations reduced *F*. *nucleatum*–mediated tumor progression, with a marked decrease in both tumor cell proliferation and *F*. *nucleatum* distribution in the crypts. Although we identified several *F*. *nucleatum* proteins potentially involved in this interaction, future research is needed to determine which proteins act as virulence factors versus those with mere surface adhesion functions. Recent studies of the human *LY6A* gene sequence have revealed aberrant *LY6A* expression in pituitary tumors, but not in normal pituitary tissues, highlighting its potential as a therapeutic target for CRC (31). Our research deepens the understanding of the bidirectional communication between host and microbiota and highlights the important role *F*. *nucleatum* infection plays in the generation of tumor initiation stem cells in human and mouse models. Additionally, we validated higher expression levels of colonic LY6A in mice treated with *F*. *nucleatum*, which consequently attracted an increased enrichment of *F*. *nucleatum*, promoting the transformation of LY6A^+^ RSCs into tumor stem cells. This interaction represents a perpetually deteriorating feedback mechanism.

Recent studies have demonstrated elevated ribosomal DNA (rDNA) transcription and enhanced protein synthesis, characteristic of active stem cell populations, within tumor tissues. These findings suggest that key genes associated with ribosomal function and protein production may drive lineage transitions and activate critical intracellular signaling pathways within the tumor microenvironment (25). One such gene, *RPS14*, is a component of the 40S small ribosomal subunit and plays a pivotal role in ribosome biogenesis and protein translation. Somatic heterozygous loss of RPS14 (uS11) has been implicated in disorders such as 5q syndrome and Diamond-Blackfan anemia (DBA), both of which present with a tumor-prone phenotype (24, 32). Additionally, RPS14 haploinsufficiency leads to a p53-dependent defect in erythroid differentiation and a loss of hematopoietic stem cell (HSC) quiescence (33). Despite the extensive literature on RPS14 in hematologic malignancies, its role in CRC remains underexplored. Our findings highlight the critical role of *F*. *nucleatum* in the generation of tumor-initiating stem cells and emphasize the bidirectional communication between host and microbiota. This work provides diverse insights into how *F*. *nucleatum* drives the transformation of LY6A^+^ stem cells into tumor stem cells through the upregulation of RPS14, a ribosomal protein linked to abnormal stem cell proliferation and tumorigenesis. Furthermore, we observed that the upregulation of ERK1/2 phosphorylation in RPS14^+^ regions (data not shown), along with increased KI67 expression, promoted cell proliferation, reinforcing the role of RPS14 in tumor development.

Moreover, previous research has shown that LY6A^+^ cells are activated during intestinal inflammation, parasitic infections, and tissue injury, primarily through the YAP signaling pathway, which regulates regeneration to counteract inflammation and injury. Our findings reveal a dual role for LY6A^+^ cells in response to injury and infection. Specifically, during *F*. *nucleatum* infection, activation of RPS14 in LY6A^+^ cells appeared to trigger a shift toward abnormal hyperproliferation, transforming these regenerative cells into tumor-initiating stem cells. We believe this discovery is meaningful on multiple levels. It not only strengthens the evidence for LY6A^+^ stem cells as tumor-initiating cells but also identifies RPS14 as a new signaling molecule driving this transformation. Additionally, it provides further insight into the association between *F*. *nucleatum* infection and tumor-initiating stem cells in CRC.

Proteomics analysis following *F*. *nucleatum* intervention revealed that upregulated proteins were clearly enriched in ribosomal and pathogen infection–related pathways. Importantly, the gut-specific KO of LY6A and the ablation of LY6A^+^ cells resulted in a marked reduction in RPS14 expression, further highlighting the importance of this molecule in *F*. *nucleatum*–mediated tumorigenesis. However, the precise mechanism by which *F*. *nucleatum* binding to LY6A leads to RPS14 upregulation remains unclear. It is still unknown whether LY6A itself activates downstream signaling pathways or merely serves as an adhesion receptor for *F*. *nucleatum*. Further research is needed to determine whether *F*. *nucleatum* directly triggers these pathways or if additional host factors are involved in this process.

In summary, our study reveals that *F*. *nucleatum* contributed to the initiation of tumor stem cells and highlights the complex spatial and temporal dimensions of the *F*. *nucleatum*–CRC association. These findings not only elucidate the pathogenic mechanisms by which *F*. *nucleatu*m promotes CRC but also provide a foundation for future therapeutic strategies targeting microbial interactions with stem cells to prevent CRC initiation and progression.

## Methods

### Sex as a biological variable.

Our study examined male and female animals, and similar findings are reported for both sexes. Human samples were obtained from both male and female participants.

### Study cohorts.

Our analysis included 3 distinct patient cohorts (pathological details are provided in Supplemental Table 2). For cohort 1, comprising 19 patients, FISH was performed for *F*. *nucleatum* distribution in cancerous and paracancerous tissues. For cohort 2, which included 62 patients with CRC, preoperative fecal samples from these patients were analyzed via metagenomic sequencing, while tissue samples were used for transcriptome sequencing. For cohort 3, comprising 6 patients, single-cell sequencing of both tumor and matched normal tissue samples was performed. Resected gut specimens were obtained during surgery and immediately sectioned into 3 parts, followed by preservation in liquid nitrogen, tissue storage solution, or fixation solution, according to the requirements of each experimental procedure.

### Animal models.

The following mouse strains were obtained from GemPharmatech: JGpt-H11em1Cin (CAG-LoxP-ZsGreen-Stop-LoxP-tdTomato) B6-G/R (strain no. T006163), Ly6a-P2A-iCre (strain no. T006978), Rosa26-SA-IRES-LoxP-ZsGreen-Stop-LoxP-DTA (strain no. T009408), and C57BL/6JGpt-H11em1Cin (pVil1-CreERT2)/Gpt (strain no. T004829). *Ly6a^fl/fl^* and *Rps14^fl/fl^* mice were purchased from Cyagen Biosciences. To trace the lineage of cells derived from LY6A-expressing cells, we generated *Ly6a^CreERT2^ H11^G/R^* mice by breeding *Ly6a^CreERT2^* mice with JGpt-H11em1Cin (CAG-LoxP-ZsGreen-Stop-LoxP-tdTomato) mice, enabling us to track the fate of LY6A^+^ cells following tamoxifen administration. For selective depletion of LY6A^+^ cells, we generated *Ly6a-DTR* mice by crossing *Ly6a^CreERT2^* mice with Rosa26-SA-IRES-LoxP-ZsGreen-Stop-LoxP-DTA mice. For conditional KO of *Ly6a* in the gut, we crossed *Ly6a^fl/fl^* mice with *Vil1^CreERT2^* mice. To knock out *Rps14* specifically in LY6A^+^ stem cells, we bred *Rps1*4*^fl/fl^* mice with *Ly6a^CreERT2^* mice. We generated *Ly6a^CreERT2^ H11^G/R^ Rps14^fl/fl^* mice by breeding *Ly6a^CreERT2^ H11^G/2^* mice with *Ly6a^CreERT2^ Rps14^fl/fl^* mice.

All animals were housed in autoclaved microisolator cages and provided sterile drinking water and chow ad libitum at the FUSCC animal center. Male mice aged 6–8 weeks were used for the experiments. Mice were administered an intraperitoneal injection of tamoxifen (MilliporeSigma, T5648) (3 mg/20 g BW) resuspended in corn oil (10 mg/mL). The number and timing of injections was tailored to the specific mouse strain and experimental design. For BrdU (MilliporeSigma, 32160405) labeling experiments, mice received a single intraperitoneal injection of BrdU (1 mg in 200 μL) twelve to 24 hours prior to tissue sampling.

### Cell cultures.

CT26 cells were cultured in RPMI 1640 medium, and HEK293 cells were cultured in DMEM with 10% FBS (Gibco, Thermo Fisher Scientific) at 37°C in a humidified 5% CO_2_ atmosphere. All cells were authenticated and tested for mycoplasma.

### F. nucleatum quantification.

To detect *F*. *nucleatum* in fecal and tissue samples from mice after gavage, fresh fecal pellets and colon were collected aseptically. Approximately 100–200 mg fecal matter or 25–50 mg tissue were processed for DNA extraction. Colonic tissues were digested in PBS containing an enzymatic cocktail of mutanolysin (250 U/mL) and lysozyme (1 mg/mL) (MilliporeSigma) at 37°C for 1 hour; total genomic DNA was isolated using QIAamp DNA Mini Kit (QIAGEN). Genomic DNA from stool was extracted using a DNeasy PowerSoil kit (QIAGEN). Quantitative PCR (qPCR) was performed for detection, using primers specific to *F*. *nucleatum* (targeting the nusG gene). The qPCR reactions were prepared in a 20 μL total volume containing 10 μL 2× qPCR master mix, 0.5 μL forward primer, 0.5 μL reverse primer, 2 μL extracted DNA, and 7 μL nuclease-free water. Amplification was performed with the following conditions: initial denaturation at 95°C for 10 minutes, followed by 40 cycles at 95°C for 15 seconds and 60°C for 1 minute. The Ct values were analyzed, with samples considered positive for *F*. *nucleatum* if the Ct value was less than 35. For PCR detection, DNA samples were loaded into an electrophoresis tank and run at 120 V for 30 minutes, followed by gel imaging. Optionally, viable *F*. *nucleatum* was detected by plating homogenized samples on blood agar plates and incubating under anaerobic conditions at 37°C for 5–7 days.

### AOM/DSS mouse model.

In the AOM/DSS mocel (MilliporeSigma, A5486 and MP Biomedicals, MFCD00081551, respectively), 6- to 7-week-old male mice were pretreated with metronidazole (2 mg/mL) (MilliporeSigma) in the drinking water for 5 consecutive days to ensure the consistency of regular microbiota and facilitate *F*. *nucleatum* colonization. After this treatment, mice were administered 1 × 10^9^ CFU *F*. *nucleatum* (or PBS as a control, PBS vs. PBS suspension of *F*. *nucleatum* pellet) by daily gavage. Following *F*. *nucleatum* administration, a single intraperitoneal injection of the carcinogen AOM (10 mg/kg body weight) was given. Seven days after the injection, 2% DSS was added to the drinking water for 7 consecutive days, after which regular drinking water was provided. Two week later, the mice received a second cycle of 2% DSS in the drinking water for another 7 days. This process was repeated for a total of 3 cycles. *F*. *nucleatum* or PBS administration continued for a total of 14 weeks.

To investigate the effect of treatment on *F*. *nucleatum*–induced gut tumor progression, mice were anesthetized and sacrificed at designated time points for tumor evaluation and histopathological analysis. The colon tissues were longitudinally opened, and tumors were measured. Tumors were categorized into 3 groups: small (<1 mm), medium (1 mm ≤ size ≤ 2 mm), and large (>2 mm). The tumor load was calculated using the following formula: tumor load = (number of small tumors) × 1 + (number of medium tumors) × 2 + (number of large tumors) × 3.

### FISH assay.

Localization of *F*. *nucleatum* was examined using a *Fusobacterium*-specific FISH probe (5′-CGCAATACAGAGTTGAGCCCTGC-3′), whereas total bacteria were detected using the universal bacterial FISH probe EUB338 (5′-GCTGCCTCCCGTAGGAGT-3′) with a bacteria FISH kit (Guangzhou Exon Biotechology Co. Ltd., focofish D-0016). Briefly, tissue sections were dehydrated and incubated with the target probes at 45°C in a dark humidified chamber for 45 minutes. The samples were then counterstained with the target antibody or DAPI. Immunostained slides were imaged with an Olympus FV300 or Nikon Ti2 microscope. Data were analyzed with ImageJ software (NIH).

### Bacterial strain and culture conditions.

The *F*. *nucleatum* strain used in this study was obtained from the American Type Culture Collection (ATCC) (*F. nucleatum* subsp. ATCC 25586) and cultured as described previously (21). Briefly, *F*. *nucleatum* was grown on Columbia blood agar supplemented with 5 μg/mL haemin, 5% defibrinated sheep blood, and 1 μg/mL vitamin K1 (MilliporeSigma) in an anaerobic glove box (85% N_2_, 10% H_2_, 5% CO_2_) at 37°C.

### Gavage procedure.

For animal experiments, *F*. *nucleatum* was cultured anaerobically overnight. The bacterial culture was centrifuged at 4,000*g* for 10 minutes, and the pellet was washed twice with sterile anaerobic PBS. Bacterial viability was detected by microscopic examination, and CFU were quantified. The bacterial suspension was adjusted to 10^10^ cells/mL and used for subsequent animal treatments.

### Intestinal tissue staining.

For fluorescent protein staining, colon tissues were fixed in 4% paraformaldehyde (PFA) at 4°C overnight, washed with PBS, embedded in OCT, and sectioned using a vibratome. Sections were permeabilized in PBS with 3% BSA and 1% Triton X-100 before staining.

For H&E staining, mouse colons were fixed in 4% PFA and processed for paraffin embedment. Sections of 7 μm thickness were stained with H&E and imaged using a Nikon Eclipse 90i microscope.

For IF, paraffin-embedded sections were rehydrated, antigen retrieval was performed, and sections were incubated with primary antibodies overnight. Sections were incubated with secondary antibodies for 1 hour, and DAPI was used to visualize nuclei. Images were captured using a Olympus FV300 or a Nikon Ti2 microscope.

The following primary antibodies were used at the indicated ratios for IF staining: rat anti-LY6A (1:50) (R&D Systems,MAB1226); mouse anti-RPS14 (1:250) (Absin,abs154574); mouse anti-BrdU (1:200) (MilliporeSigma, B8434); rabbit anti–*Fusobacterium nucleatum* 25586 (1:1,000) (Diatheva, ANT0084); and rabbit anti-Ki67 monoclonal antibody (1:100) (Cell Signaling Technology, 9129S). The following secondary antibodies were from Invitrogen (Thermo Fisher Scientific): donkey anti–rabbit IgG (H+L) highly cross-adsorbed secondary antibody, Alexa Fluor 647 (1:1,000) (A-31573); goat anti–mouse IgG (H+L) cross-adsorbed secondary antibody, Alexa Fluor 647 (1:1,000) (A-21235); goat anti–rat IgG (H+L) cross-adsorbed secondary antibody, Alexa Fluor 647(1:1,000) (A21247); donkey anti–mouse IgG (H+L) highly cross-adsorbed secondary antibody, Alexa Fluor 488 (1:1,000) (A 21202); goat anti–mouse IgG (H+L) cross-adsorbed secondary antibody; cyanine 3 (1:1,000) (A10521); goat anti–rabbit IgG (H+L) cross-adsorbed secondary antibody, Alexa Fluor 488 (1:1,000) (A11008); and goat anti–rabbit IgG (H+L) cross-adsorbed secondary antibody, cyanine 3 (1:1,000) (A10520). DAPI solution (1:5,000) (MilliporeSigma, D9542) was used to counterstain nuclei.

### Colon organoid culturing.

The colon was excised and rinsed with PBS, and the colonic mucosa was scraped from the muscle layer. The minced tissue was digested using gentle cell dissociation reagent (GCDR) (STEMCELL Technologies, 100-0485) and filtered to isolate crypts or cell clusters. Cells were resuspended in cold Matrigel (Corning, 356231) and then plated and incubated at 37°C to allow solidification. Prewarmed IntestiCult Organoid Growth Medium (STEMCELL Technologies, 6005) was added, followed by incubation at 37°C with 5% CO_2_. The medium was changed every 2–3 days, and organoids formed within 3–4 days. For passaging, organoids were harvested, fragmented, and reseeded in fresh Matrigel. For tumoroid generation, intestinal adenoma cells from 3 tumors from PBS- or *F*. *nucleatum*–treated *Ly6a^CreER2^ H11^G/R^* mice were isolated by lysis in 2 mg/mL collagenase type V (MilliporeSigma, C9263) for 30 minutes. Single cells were embedded in Matrigel and seeded onto 24-well plates. The same organoid culture medium as described above was used. To prevent anoikis, 10 μM Y-27632 (Selleck Chemicals, S1049) was added during the first 2 days after isolation, culturing, or passaging.

### Cell sorting.

To establish LY6A-tdTomato^+^ stem cell–derived organoids, lineage tracing was induced over 7 days or 12 months in at least 3 *Ly6a^CreER2^ H11^G/R^* mice as previously described above. The mice were sacrificed, and the colons or tumor were isolated. Colonic tissue and tumor were digested as described above. For the colonic tissue, after incubation, the samples were vigorously shaken to dissociate the tissue. The tissue fragments were filtered through a 70 μm filter, and the supernatant containing the crypts was collected. The crypts were then washed and resuspended in a cell dissociation solution containing TrypLE Express (Gibco, Thermo Fisher Scientific, 12604021). Following a 15-minute incubation at 37°C, cells were washed in Advanced DMEM/F12 medium (STEMCELL Technologies, 36254) containing 1× penicillin-streptomycin. After an additional wash, the cells were resuspended in Advanced DMEM/F12 medium supplemented with N2 and B27 and then filtered through a 40 μm filter. The tdTomato^+^ cells were sorted by flow cytometry using a BD FACSAria II flow cytometer.

### F. nucleatum attachment assay.

CT26 or HEK293 cells were cocultured with *F*. *nucleatum* at a MOI of 10 for 2 hours under aerobic conditions. Following coculturing, the cells were washed 5 times with PBS to remove unattached bacteria and then lysed for 5 minutes with 500 μL ice-cold 1× Triton X-100. The lysates were serially diluted (10-, 100-, and 1,000-fold) and plated on blood agar plates. After 1 week’s incubation at 37°C under aerobic conditions, CFU were counted to quantify bacterial attachment.

### F. nucleatum membrane protein extraction.

For the preparation of *F*. *nucleatum* surface proteins, the bacteria were cultured at 37°C under aerobic conditions for 24 hours. The bacterial cells were harvested by centrifugation at 3,681*g* for 5 minutes, and the supernatant was discarded. The bacterial pellets were then washed with ice-cold PBS, and the membrane proteins were extracted using the Membrane and Cytosol Protein Extraction Kit (Beyotime, P0033) according to the manufacturer’s instructions.

### Flag IP assay and protein identification.

For the IP assay, transfected cells were washed 3 times with PBS and lysed using RIPA buffer (150 mM NaCl, 0.1% SDS, 1% NP40, 0.5% sodium deoxycholate, 1 mM EDTA, 50 mM Tris, pH 8.0) for 30 minutes on ice. Cell lysates were cleared by centrifugation at 14,000*g* for 10 minutes. The extracted Flag-tagged LY6A proteins were incubated with Flag magnetic beads (MilliporeSigma, M8823) for 4 hours at 4°C. After washing 3 times with IP buffer on a magnetic bead, the Flag-bound LY6A proteins were incubated overnight at 4°C with *F*. *nucleatum* surface proteins. The samples were then washed 5 times with IP buffer. The LY6A–*F*. *nucleatum*–interacting proteins were eluted by heating in 50 μL 1× loading buffer at 100°C. Eluted proteins were separated by SDS-PAGE, and the entire gel was subjected to silver staining by ProteoSilver Plus kit (MilliporeSigma, PROTSIL1) according to the manufacturer’s instructions. The corresponding bands detected in the silver-stained gel were excised for protein identification via mass spectrometry.

### Western blotting.

Proteins from cell lines/organoids or tissues were extracted using RIPA (50 mM tris-HCl [pH 8.0], 150 mM NaCl, 0.1% SDS, 0.15% Na-deoxycholate, 1% NP-40, and 2 mM EDTA [pH 8.0]) containing protease inhibitors (MedChemExpress, 317717) and the phosphatase inhibitor PhosSTOP (Roche, 04906845001), and colon or tumor tissues were extracted using Protein Extraction Reagent (Thermo Fisher Scientific) following the manufacturer’s instructions. Protein concentrations were measured using a Pierce BCA Protein Assay Kit (Thermo Fisher Scientific). Equal amounts of protein lysates were separated by SDS-PAGE and transferred onto PVDF membranes (MilliporeSigma). After blocking with 5% BSA at room temperature for 2 hours, the membranes were incubated overnight at 4°C with the following specific primary antibodies: rabbit anti-LY6A (1:1,000) (Signalway Antibody, 37285); mouse anti-RPS14 (1:1,000) (Abclonal, A6727); and anti-FLAG M2 antibody (1:1,000) (Cell Signaling Technology,14793). HRP-conjugated anti–mouse IgG (Cell Signaling Technology,7076) or anti–rabbit IgG secondary antibodies (Cell Signaling Technology,7074) were used for detection. The membranes were then incubated with HRP-conjugated secondary antibodies for 1 hour at room temperature. Protein bands were visualized using the Pierce ECL detection system (Thermo Fisher Scientific). β-Actin was used as the endogenous control.

### Lentivirus shRNA construction and transfection.

Replication-deficient lentiviral particles were produced by transfecting mycoplasma-free HEK293T cells with Lipo293F Transfection Reagent (Beyotime, C0518) and a mixture of the following plasmids: packaging vector psPAX2 (Addgene plasmid 12260) and envelope vector pMD2.G (Addgene plasmid 12259). Lentiviral particles were used to infect target cells. Seventy-two hours after infection, the cells were selected with 10 μg/mL puromycin for 7 days.

### Statistics.

Statistical analyses were performed using GraphPad Prism 6 (GraphPad Software). For experiments comparing 2 groups, unless otherwise specified, the differences between means were analyzed using a 2-tailed Student’s *t* test. For experiments involving 3 or more groups, 1-way or 2-way ANOVA was used, followed by post hoc Tukey’s or Dunnett’s test for multiple comparisons. A *P* value of less than 0.05 was considered significant, and the data are presented as the mean ± SEM.

### Study approval.

All samples were obtained from the FUSCC and consist of archived research materials from patients who provided written, informed consent. The patient study was conducted in accordance with the principles of the Declaration of Helsinki and approved by the ethics committee of the FUSCC (approval ID 1911210-4). Mouse studies were approved by the research ethics Committee of the FUSCC. All mouse studies were carried out in accordance with the requirements of the Animal Research Committee of Fudan University regarding the care and use of experimental animals in research (FUSCC-IACUC-S20210410).

### Data availability.

The omics datasets generated in this study are available at the National Genomics Data Center (https://ngdc.cncb.ac.cn). Accession numbers are as follows: HRA005038 (metagenomics data; https://ngdc.cncb.ac.cn/search/specific?db=hra&q=HRA005038); HRA005810 (RNA-Seq data; https://ngdc.cncb.ac.cn/gsa-human/browse/HRA005810); and PRJCA020855 (single-cell sequencing data; https://ngdc.cncb.ac.cn/search/specific?db=hra&q=PRJCA020855). This article does not report any original code. All data supporting the findings of this study are available within the article and its supplemental material, including the Supporting Data Values file.

## Author contributions

QW and YM were responsible for the study concept and design. YG and YY provided specimens. YG and JL acquired clinical data. QW, TH, YG, and JL analyzed and interpreted data and performed statistical analyses. TH, QZ, YZ, YJ, XD, and FG conducted animal experiments. QW, XD, YJ, TH, FG, and PZ conducted organoid and cell experiments. QW, YZ, and TH performed IF experiments. ZC prepared *F*. *nucleatum* for gavage. YM, QW, and TH wrote the manuscript.

## Supplementary Material

Supplemental data

Unedited blot and gel images

Supplemental table 1

Supplemental table 2

Supplemental table 3

Supporting data values

## Version 1 12/10/2024

In-Press Preview

## Version 2 02/03/2025

Electronic publication
