# Supplementary material for: Fusobacterium nucleatum promotes colorectal cancer through neogenesis of tumor stem cells
Source: J Clin Invest. 2025 Feb 3;135(3):e181595. doi: 10.1172/JCI181595 (PMC11785920; doi:10.1172/JCI181595)
Supplement: Supplemental data [file jci-135-181595-s184.pdf]

Supplemental Figure 1

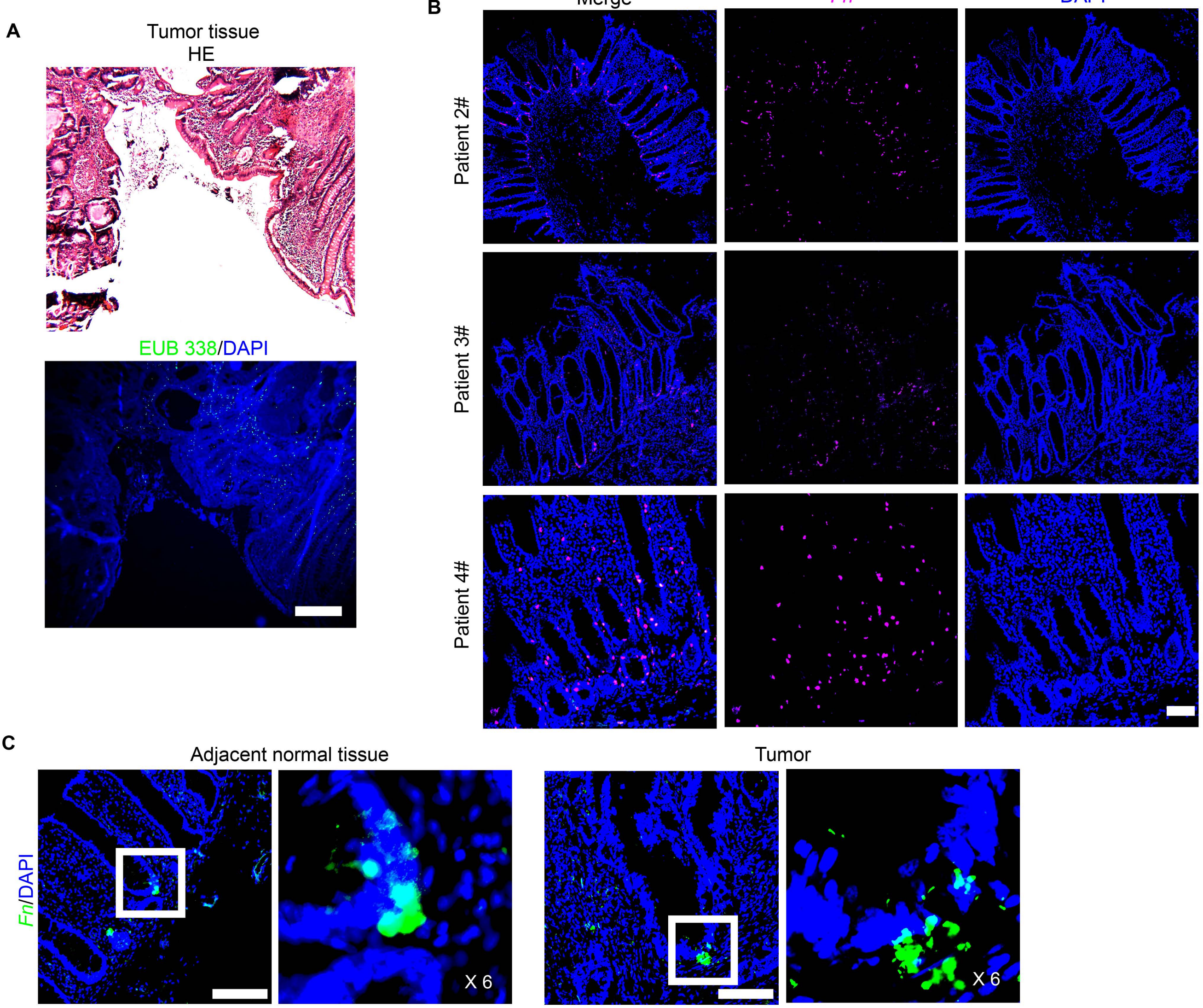

Supplemental Figure 2

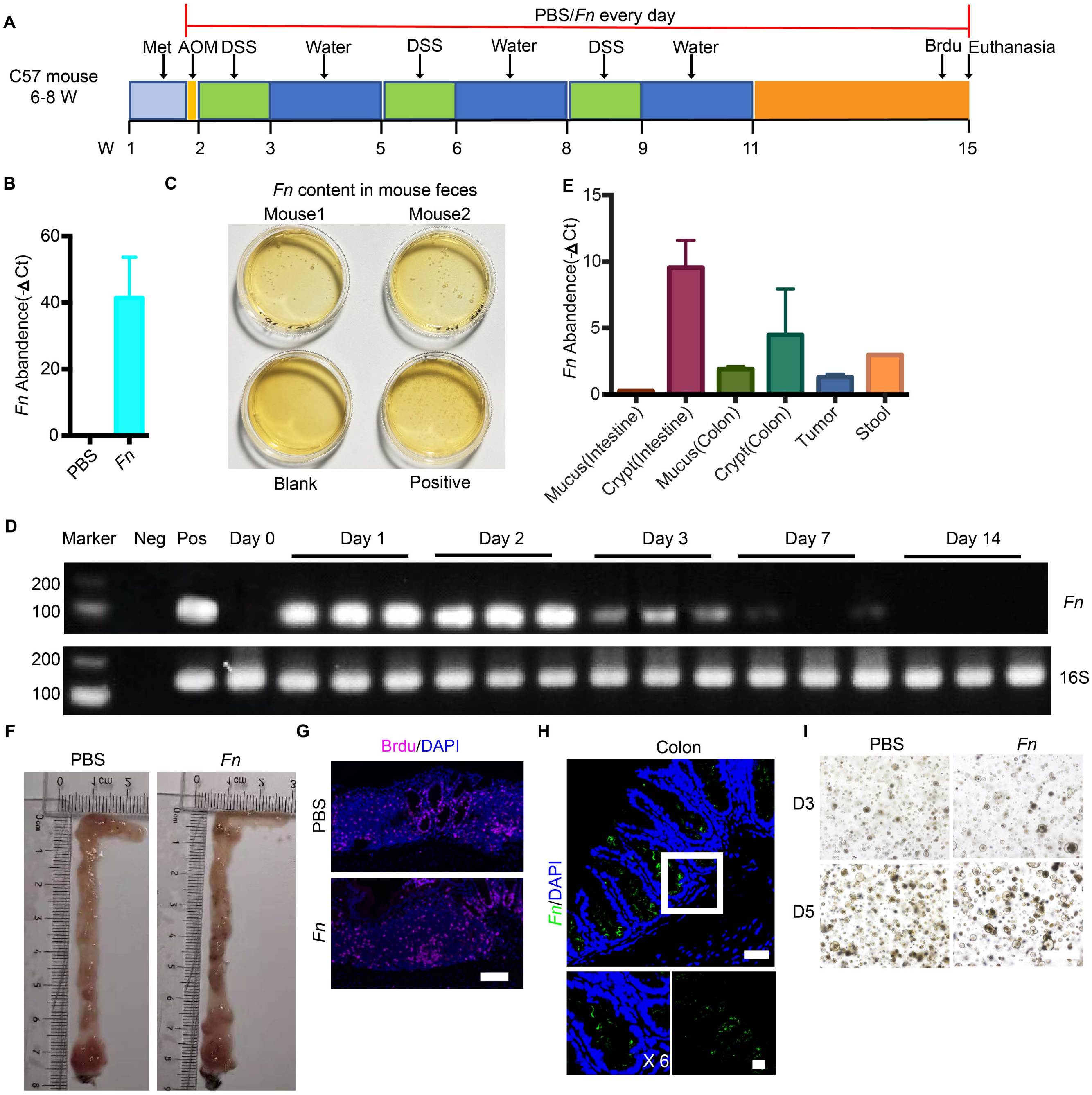

Supplemental Figure 3

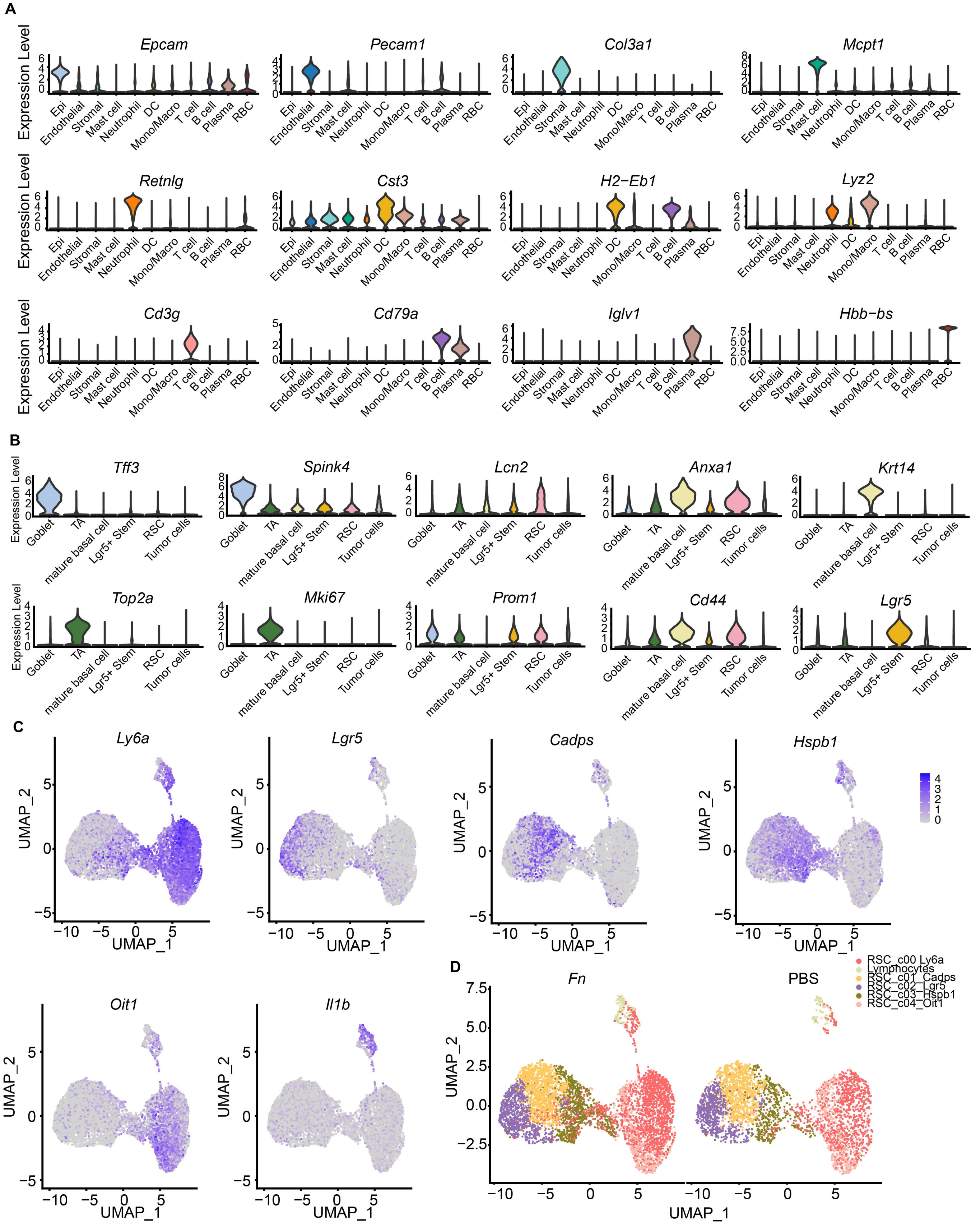

Supplemental Figure 4

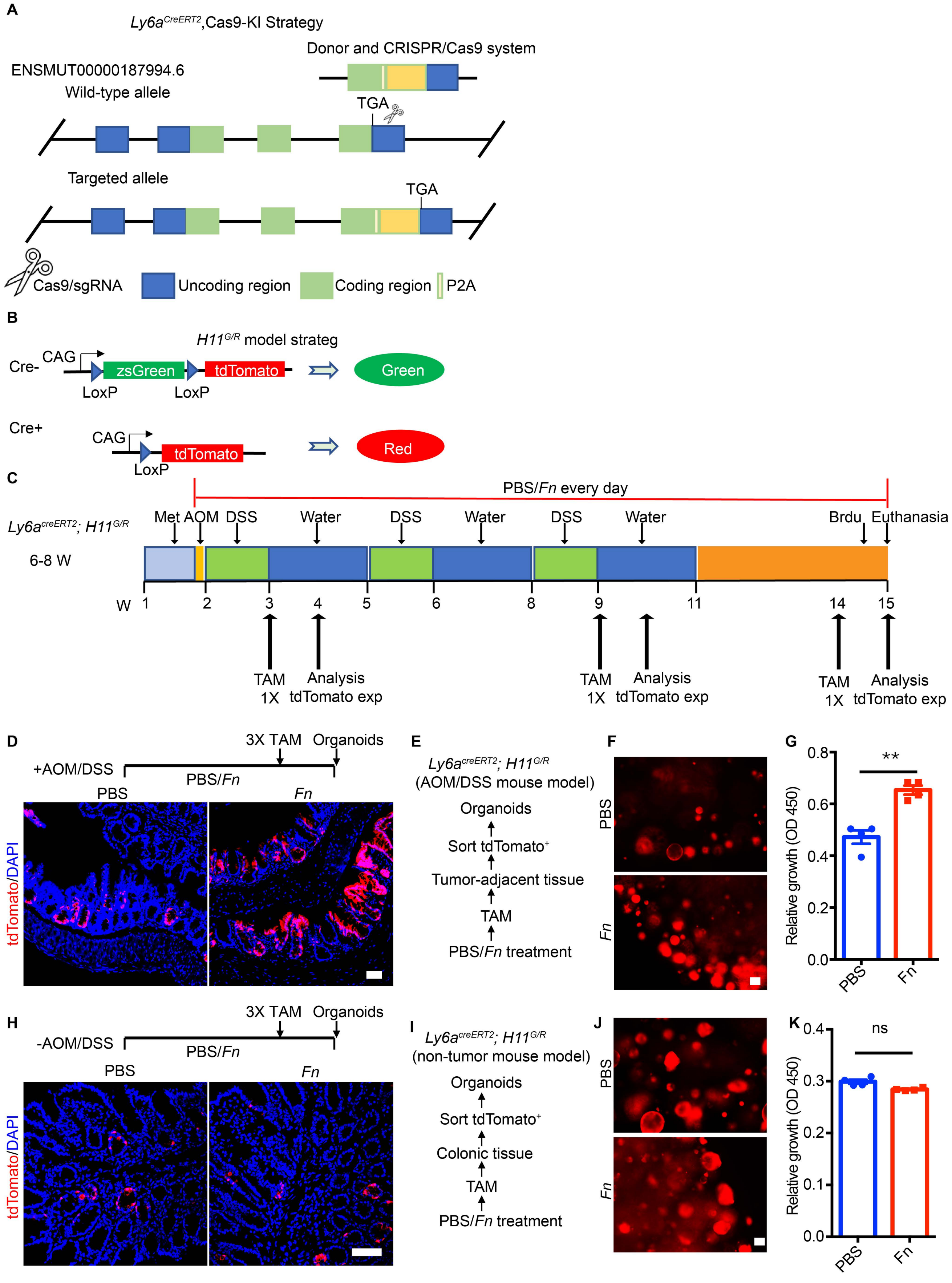

Supplemental Figure 5

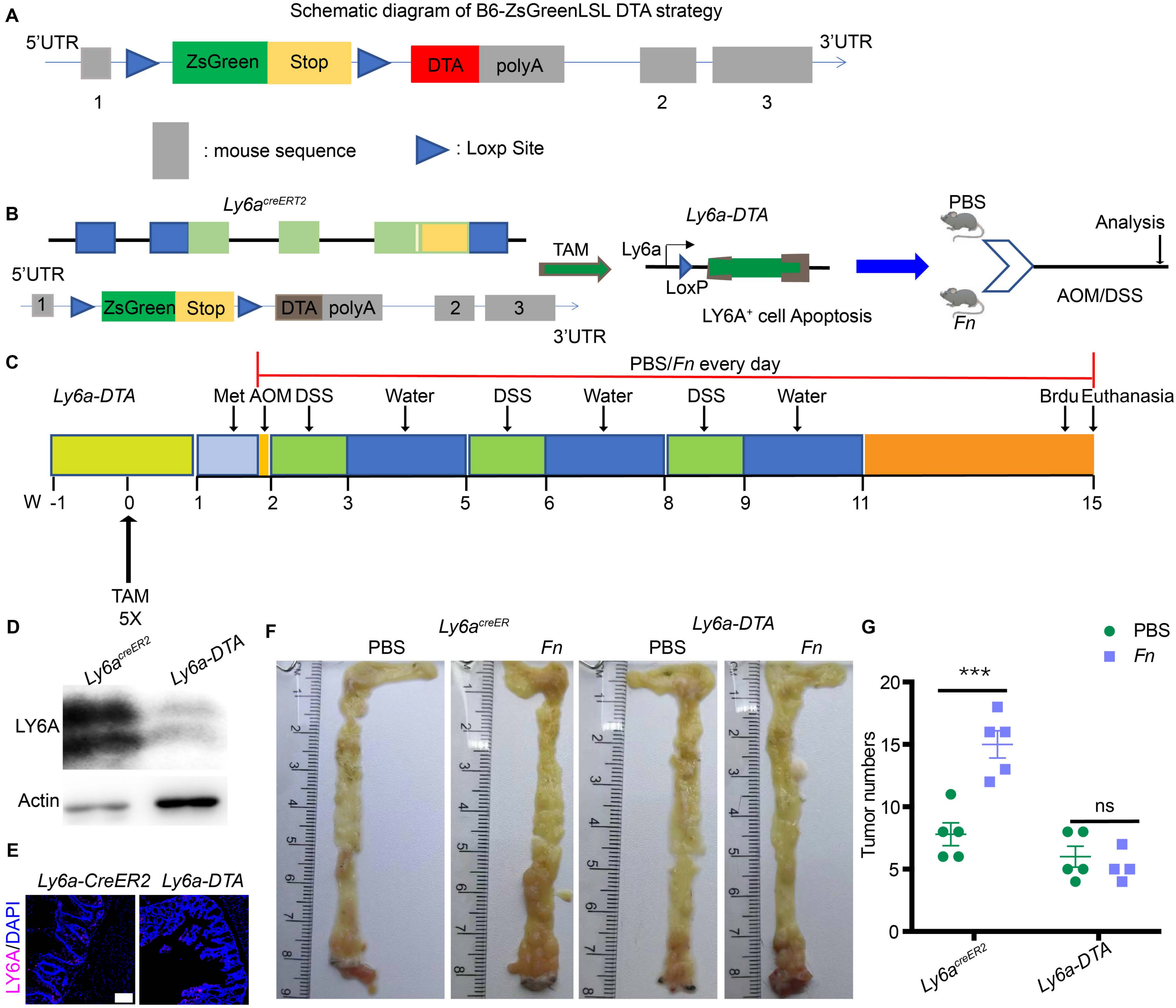

Supplemental Figure 6

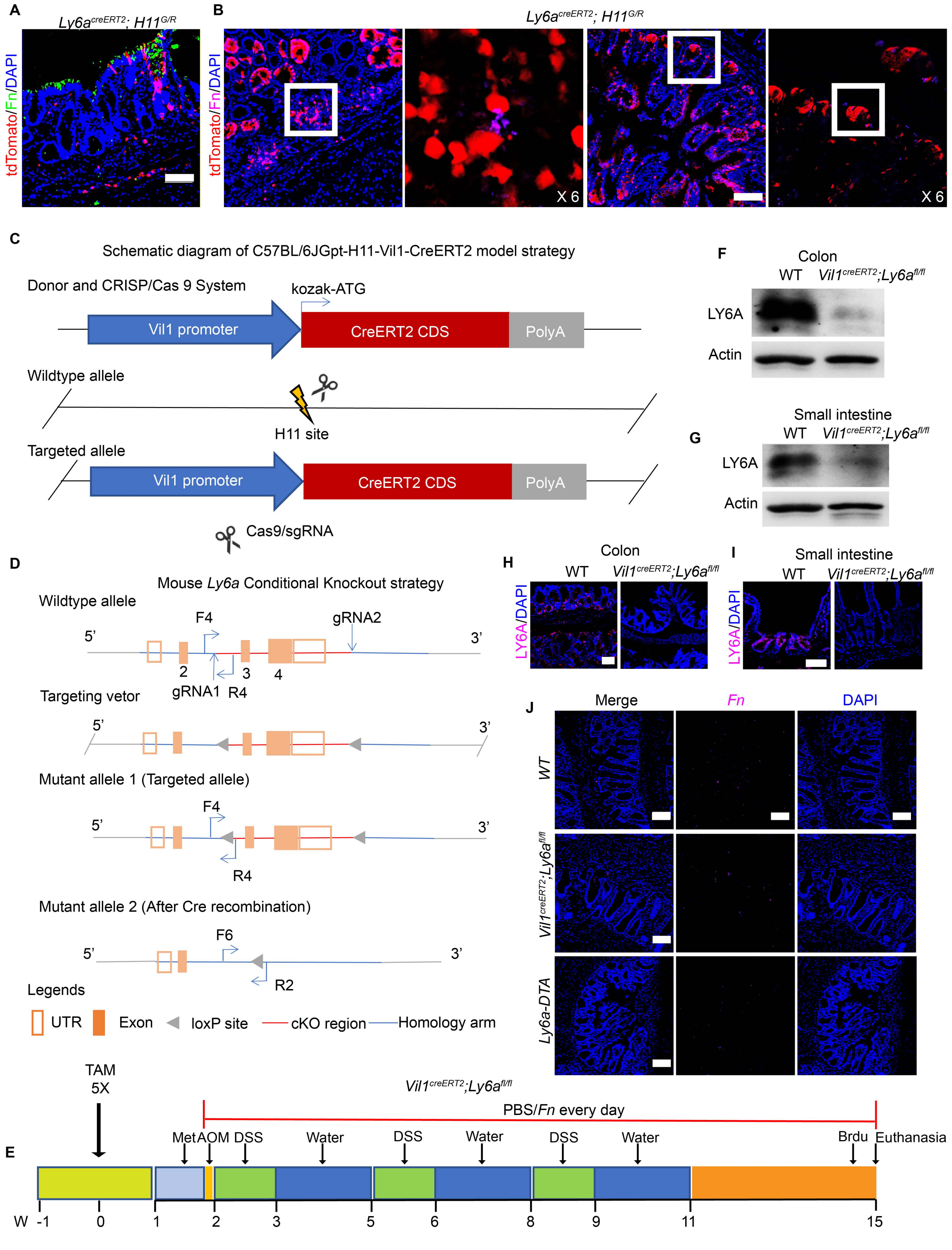

Supplemental Figure 7

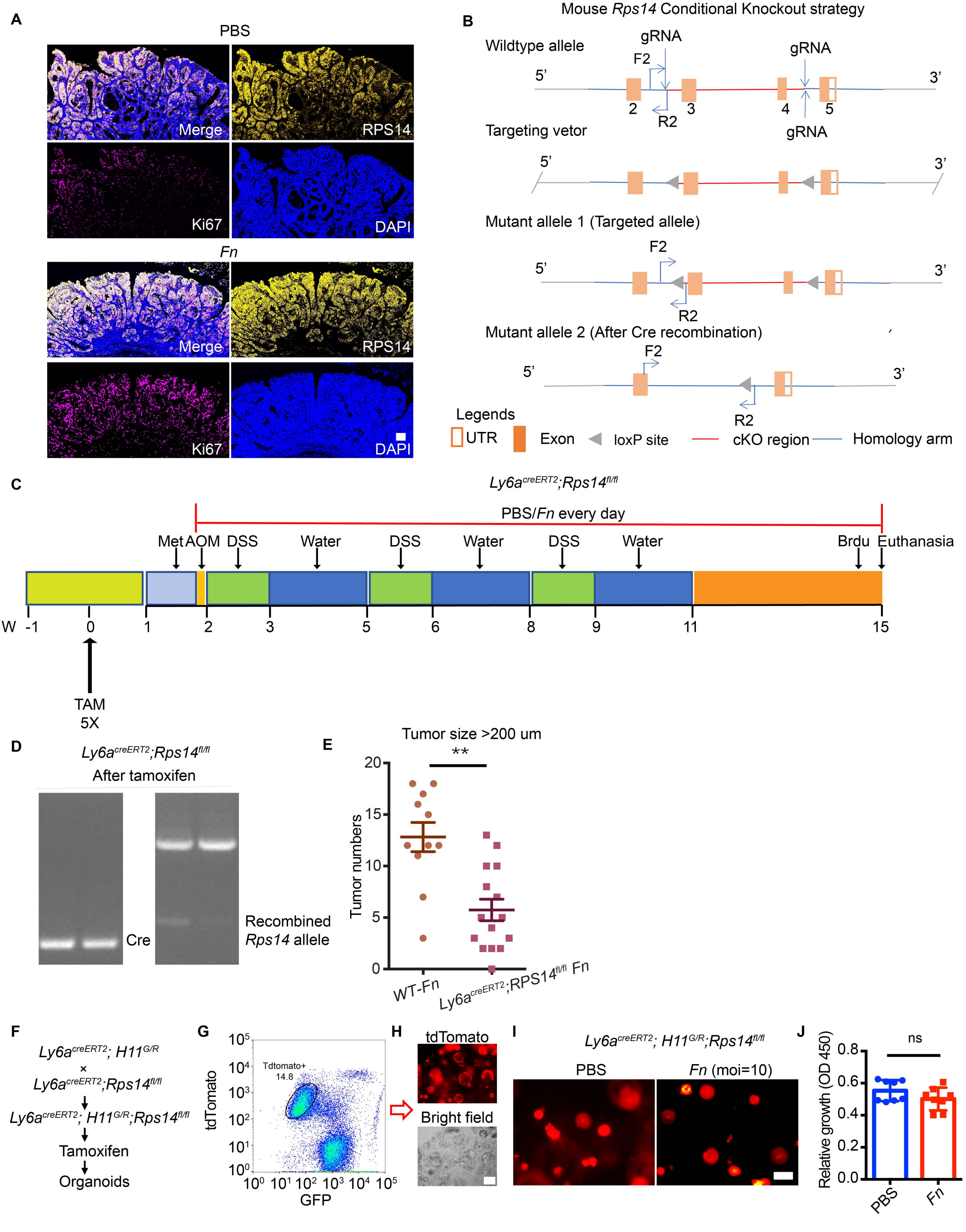

Supplemental Figure 8

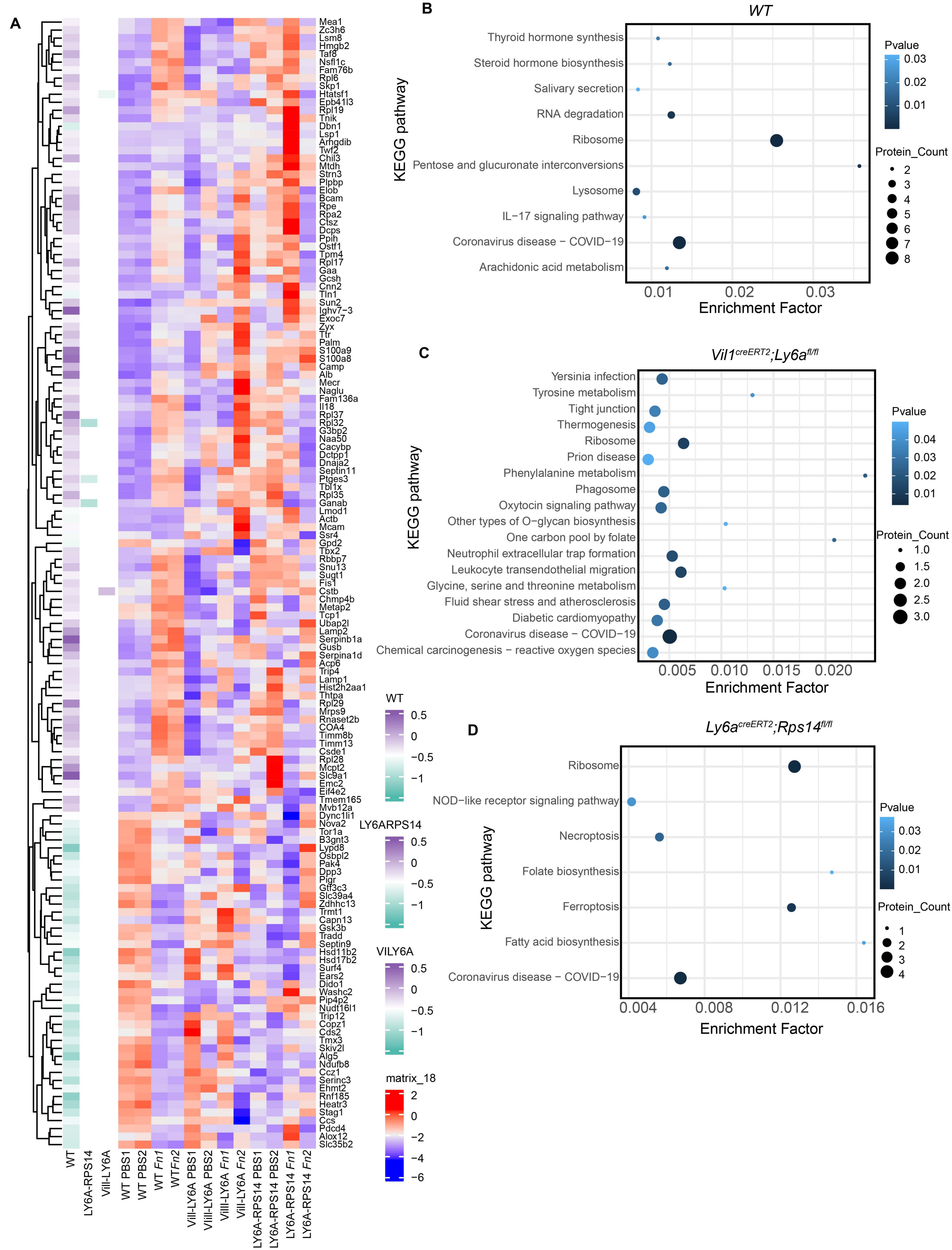

Supplemental Figure 9

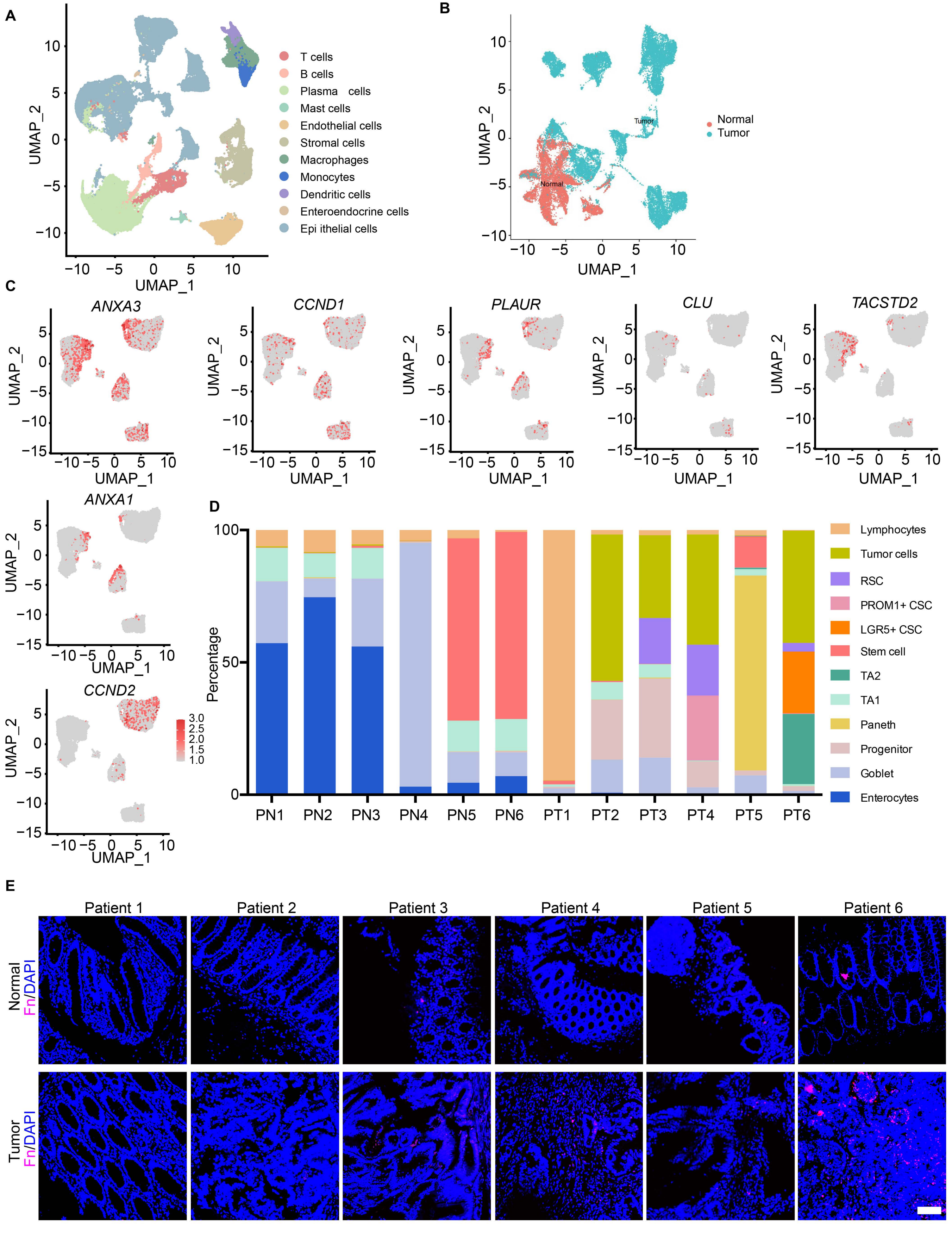

Supplemental information

Fusobacterium Nucleatum Promotes Colorectal Cancer through Neogenesis of Tumor Stem Cells

Qinying Wang[1,2,3], Tingting Hu[1,3], Qinyuan Zhang[1,3], Yichi Zhang[1,3], Xiaoxu Dong[1,3],Yutao Jin[1,3], Jinming Li[1,3], Yangyang Guo[1,3], Fanying Guo[1,3], Ziying Chen[1,3], Peijie Zhong[1,3] Yongzhi Yang[1,3] and Yanlei Ma[1,3*].

[1] Department of Colorectal Surgery, Fudan University Shanghai Cancer Center, Shanghai, China

[2] Department of Cancer Institute, Fudan University Shanghai Cancer Center, Shanghai, China

[3] Department of Oncology, Shanghai Medical College, Fudan University, Shanghai, China

Method Details

*DNA Library Construction and Metagenomic Sequencing***.**

Sequencing libraries were constructed using the TruSeq Nano DNA LT Library Preparation Kit (Illumina). DNA was fragmented by dsDNA Fragmentase (NEB) and incubated at 37°C for 30 minutes. Library construction began with fragmented cDNA. Blunt-end DNA fragments were generated through fill-in reactions and exonuclease activity. Sample purification beads were used for size selection. An A-base was then added to the blunt ends of the DNA strands, preparing them for ligation to indexed adapters. Each adapter contained a T-base overhang for ligation to the A-tailed fragmented DNA and included all necessary sequencing primer hybridization sites for single, paired-end, and indexed reads. Single- or dual-index adapters were ligated to the fragments, and the ligated products were amplified via PCR. After purification, quantification, and quality control, high-throughput sequencing was carried out on the NovaSeq6000 platform (Illumina) following the manufacturer's instructions.

Tissue dissociation, single-cell suspensions preparation, and bioinformatics analysis.

*Preparation of mouse samples***.**

A sterile, RNase-free culture dish containing calcium- and magnesium-free 1× PBS was placed on ice. Tissue was transferred into the dish, cut into 0.5 mm^2 pieces, and washed with 1× PBS to remove non-target tissues, such as blood and fatty layers. Tissue fragments were dissociated into single cells in a dissociation solution containing 0.35% collagenase IV, 2 mg/mL papain, and 120 U/mL DNase I in a 37°C water bath, shaking at 100 RPM for 20 minutes. Digestion was stopped using 1× PBS containing 10% FBS. The resulting cell suspension was pipetted 5-10 times, filtered through a 70-30 μm cell strainer, and centrifuged at 300g for 5 minutes at 4°C. The cell pellet was

resuspended in 100 μL 1× PBS (0.04% BSA) and treated with 1 mL red blood cell lysis buffer for 2-10 minutes at room temperature or on ice. The suspension was centrifuged again and resuspended in 100 μL Dead Cell Removal MicroBeads (MACS). Dead cells were removed using the Miltenyi® Dead Cell Removal Kit, and the final cell suspension was resuspended in 50 μL 1× PBS (0.04% BSA). Cell viability was confirmed by trypan blue exclusion, requiring >85% viability. Single-cell suspensions were counted using a Countess II Automated Cell Counter and adjusted to 700-1200 cells/μL.

*Preparation of human samples***.**

Collected tumor tissues were stored in sCelLive™ Tissue Preservation Solution (Singleron Biotechnologies) and transported on ice to the Singleron lab. Tissues were dissociated into single-cell suspensions using the Singleron PythoN™ Automated Tissue Dissociator and sCelLive™ Tissue Dissociation Mix following the preset protocol. Cell viability was assessed using trypan blue and evaluated microscopically.

*Generation of single-cell seq libraries***.**

Mouse Samples

Single-cell suspensions were loaded onto the 10X Chromium system to capture 8,000 single cells following the manufacturer's instructions for the Chromium Single-Cell 3' Kit (V3) (10X Genomics). cDNA amplification and library construction were carried out according to standard protocols. Libraries were sequenced on the Illumina NovaSeq 6000 platform (paired-end, 150 bp) with a minimum depth of 20,000 reads per cell.

Human Samples

Single-cell suspensions (1 × 10^5 cells/mL) were loaded onto microfluidic devices, and scRNA-seq libraries were constructed using the GEXSCOPE® Single-Cell RNA Library Kit (Singleron Biotechnologies). Libraries were diluted to 4 ng/μL, pooled, and sequenced on the Illumina NovaSeq 6000 platform with 150 bp paired-end reads.

*Bioinformatic alignment and pre-processing of single cell RNA sequencing data***.**

Sequencing results were demultiplexed and converted to FASTQ format using Illumina's bcl2fastq software. Barcode processing and UMI counting were performed using the Cell Ranger pipeline (v3.1.0). ScRNA-seq data were aligned to the Ensembl GRCh38/GRCm38 reference genome. Cells from F. nucleatum-treated AOM/DSS mice (n = 7) and PBS-treated AOM/DSS mice (n = 5) were processed using the 10X Chromium Single Cell 3' Solution. Data were loaded into Seurat (v3.1.2) for downstream analysis. Genes expressed in fewer than three cells were excluded, and low-quality cells were filtered. Dimensionality reduction was performed using UMAP, and cell clustering was carried out using Seurat's Shared Nearest Neighbor (SNN) network.

*Dimensionality reduction and visualization***.**

Counts on the filtered matrix for each gene were normalized and scaled by using Seurat.NormalizeData(). The 3000 most highly variable genes were identified by Seurat. FindVariableGenes() and each integrated feature was centered to a mean of zero and scaled by the standard deviation with the function ScaleData(). The scaled data were used to perform principal component analysis (PCA) using the Seurat.RunPCA(). To cluster cells, 30 principal components (PCs) were selected with *resolution=1.0.* to construct a Shared Nearest Neighbor (SNN) network by using FindNeighbor() and then FindClusters() was used to calculate the overlap for each cell

and it's nearest neighbors. To further visualize the data, uniform manifold approximation and projection (UMAP) of Seurat was used to reduce the dimensionality of 132864 cells and project the cells into 2D space1 by Seurat.RunUMAP().

Nomination.

Cluster-specific expressed marker genes were identified with the Wilcoxon rank sum test (Likelihood-ratio test) with default parameters via the Seurat. FindAllMarkers() was conducted between cells from the individual cluster versus cells from all other clusters to select marker genes which are expressed in more than 10% of the cells in a cluster and an average log(Fold Change) of greater than 0.75 (Supplemental Table 3). Only genes with a significant adjusted p-value ($pv<0.05$, false discovery rate (FDR) adjusted p-value) were kept to annote each cluster.

Proteome.

(i) Protein Extraction: Tissue samples were homogenized with lysis solution and Roche cocktail. After centrifugation, supernatants were collected.

(ii) Quality Control: Protein concentrations were measured using the Bradford method. SDS-PAGE was used to verify protein integrity.

(iii) Protein Digestion: Proteins were digested with trypsin, desalted, and vacuum-dried.

(iv) Quantitative Detection: Peptides were separated by nano-LC-MS/MS and analyzed using Orbitrap Exploris™ 480 in DDA mode.

(v) Protein Identification and Quantification: MaxQuant (v2.1.4.0) was used for protein analysis, with a 1% FDR threshold. Differentially expressed proteins were identified using T-tests ($p \leq 0.05$, fold change ≥ 1.2). Enrichment analyses were performed for pathway annotation.

Quantification and Statistical Analysis.

Statistical tests were performed using GraphPad Prism 6 software. For comparisons between two groups, two-tailed Student's t-tests were used. For experiments with three or more groups, one-way or two-way ANOVA with post hoc Tukey's or Dunnett's tests were used. Statistical significance was indicated as: *= $p < 0.05$, **= $p < 0.005$ and ***= $p < 0.0005$.

Supplemental Figure Legends

Supplemental Figure 1. Distribution of *F. nucleatum* in normal and tumor tissues of human, Related to Figure 1.

(**A**) Representative H&E image of colorectal tumor tissue from a patient.

(**B**) FISH assay detecting the distribution of *F. nucleatum* (20×, Cy5-conjugated, red) in the crypts of para-cancerous tissue in humans.

(**C**) Immunofluorescence assay detecting the distribution of *F. nucleatum* in the crypts of para-cancerous and cancerous tissues in humans. The white frame indicates positive *F. nucleatum* staining.

Scale bars: 100 μm.

Supplemental Figure 2. Distribution of *F. nucleatum* in normal and tumor tissues of mouse, Related to Figure 1.

(**A**) Schematic diagram illustrating the experimental design and timeline for *F. nucleatum* and PBS treatments in AOM/DSS CRC mice model.

(**B**) Stool abundance of *F. nucleatum* as determined by qPCR.

(**C**) Stool abundance of *F. nucleatum* as determined by bacterial plate spreading experiment .

(**D**) Colonic abundance of *F. nucleatum* as determined by PCR.

(**E**) *F. nucleatum* abundance in different segments of colonic tissue (mucus, submucosa, tumor, and stool) as determined by qPCR.

(**F**) Representative images of the colon and rectum in PBS- and *F. nucleatum*-treated groups, showing tumor burden.

(**G**) Representative immunofluorescence images of $Brdu^+$ cells in colonic tumors after PBS and *F. nucleatum* intervention.

(**H**) FISH assay detecting the distribution of *F. nucleatum* (40×, FITC-conjugated, green) in the crypts of mouse colon.

(**I**) Organoid growth trends from tumors in mice treated with PBS or *F. nucleatum*.

Scale bars: 100 μm in (G-H); 1mm in (I).

Supplemental Figure 3. Single-cell analysis of the increased stem cell population in tumor tissue with/without *F. nucleatum* treatment, Related to Figure 1.

(**A**) Violin plots showing colon cell marker expression in each group.

(**B**) Violin plots showing epithelial cell marker expression in each group.

(**C**) Feature plot showing the expression of marker genes that identify RSC clusters.

(**D**) Feature plot showing the proportion of RSCs in PBS- and *F. nucleatum*-treated groups.

Supplemental Figure 4. Construction of *Ly6a*^{creER} ; *H11*$^{G/R}$ mice and detection of the activation of LY6A^{+} RSCs in both colorectal and non-colorectal cancer models, Related to Figure 2.

(**A**) The schematic diagram of *Ly6a*^{creERT2} knockin mice via CRISPR/Cas9 system. Cas9 mRNA, sgRNA and donor was co-injected into zygotes. sgRNA direct Cas9 endonuclease cleavage near stop coding (TGA) of *Ly6a* gene and create a DSB (double-strand break). Such breaks was repaired and result in P2A-iCre before stop coding (TGA) of *Ly6a* gene by homologous recombination.

(**B**) Schematic diagram of *H11*$^{G/R}$ model strategy. B6-G/R combined the green fluorescent protein ZsGreen with the red fluorescent protein tdTomato and knocked it into the mouse H11 safe harbor site at the same time. Under normal circumstances, the whole body of mice expresses ZsGreen, which can excite dazzling green light that may illuminate the shape and structure of various cells and tissues. Therefore, the whole body of mice is green at birth, which is different. However, it buried loxP sites on both sides of ZsGreen. When Cre recombinase exists, ZsGreen in the mouse genome will be deleted, and the expression of tdTomato will be turned on, which can emit a dazzling red light. Since Cre can be expressed tissue-specifically, mice turn on the red light following the expression time and location of Cre.

(**C**) Scheme of the protocol and animals. TM: 3mg/20g for each mouse.

(**D**) Representative fluorescent images of lineage-tracing experiments in the adjacent normal colonic tissue of *Ly6a*^{creERT2}*; H11*$^{G/R}$ AOM/DSS CRC mice model treated with PBS or *F. nucleatum* at 15 weeks.

(**E**) Schematic of experimental design showing tamoxifen treatment for LY6A-tdTomato expression and generate organoids.

(**F**) Representative image of organoids generated by LY6A-tdTomato^{+} cells from adjacent normal colonic tissues after PBS or *F. nucleatum* treatment.

(**G**) Organoid growth rate from adjacent normal colonic tissues of mice treated with PBS or *F. nucleatum*.

(**H**) Representative fluorescent images of lineage-tracing experiments in the colon of *Ly6a*^{creERT2}*; H11*$^{G/R}$ non-CRC mice model treated with PBS or *F. nucleatum* at 15 weeks.

(**I**) Schematic of experimental design showing tamoxifen treatment for LY6A-tdTomato expression to generate organoids.

(**J**) Representative image of organoids generated by LY6A-tdTomato^{+} cells from colon after PBS or *F. nucleatum* treatment.

(**K**) Organoid growth rate from colon of mice treated with PBS or *F. nucleatum*.

Scale bars: 100 μm.

The data are representatives and are presented as mean ± SEM of at least 3 assays. *P < .05; **P < .01.

Statistics: (G) and (K): Student's t test (two-tailed).

Supplemental Figure 5. Construction of *Ly6a-DTA* mice and detection of LY6A^{+} RSCs ablation on the progression of CRC with PBS or *F. nucleatum* treatment, Related to Figure 2.

(**A**) Schematic diagram of B6-ZsGreenLSL DTA strategy. B6/JGpt-Rosa26em1Cin (SA-IRES-Loxp-ZsGreen-stop-Loxp-DTA)/Gpt. Diphtheria toxin is secreted by pathogenic strains of Corynebacterium diphtheriae and is composed of two subunits, α and β. Subunit β is responsible for the internalisation of the toxin upon binding to its receptor. Once inside the cell, subunit α catalyses the inactivation of elongation factor 2, resulting in termination of protein synthesis and apoptosis of the target cell. Here it uses gene editing technology to develop B6-ZsGreenLSL DTA mouse. When B6-ZsGreenLSL DTA mice breed with Cre recombinase-expressing mice, the LSL will be deleted in cells which express Cre recombinase, and DTA expression will be turned on to induce specific cell ablations. Therefore, the B6-ZsGreenLSL DTA model can be used to study the specific tissues and cells developmental and function.

(**B**) Strategy for selective LY6A lineage deletion through Cre activation to induce DTA expression and selective death of LY6A^{+} cells.

(**C**) Schematic diagram showing the experimental design and timeline of *Ly6a-DTA* mice under *F. nucleatum* and PBS treatments.

(**D** and **E**) Ablation efficiency of LY6A^{+} cells in *Ly6a-DTA* mice after tamoxifen injection.

(**F**) Representative images of the colon and rectum in PBS- and *F. nucleatum*-treated *Ly6a-DTA* mice, showing tumor burden.

(**G**) Tumor number in the colon and rectum measured at the end of the study.

Scale bars: 100 μm.

The data are representatives and are presented as mean ± SEM of at least 3 assays. *P < .05; **P < .01.

Statistics: (G): Two-way ANOVA with post hoc Tukey's multiple comparisons.

Supplemental Figure 6. Specific binding of *F. nucleatum* to LY6A molecules, Related to Figure 3.

(**A**) FISH assay detecting direct contact between *F. nucleatum* and LY6A-tdTomato^{+} cells in the crypts of mouse colons after 10 weeks.

(**B**) FISH assay detecting direct contact between *F. nucleatum* and LY6A-tdTomato^{+} cells in the crypts of mouse colons after 15 weeks.

(**C**) Schematic diagram of the $Vil1^{creERT2}$ model strategy. This strain expresses $Vil1^{creERT2}$ under the control of the mouse Vil1 promoter, inducing site-specific recombination after tamoxifen administration.

(**D**) Design strategy for the $Ly6a^{fl/fl}$ conditional knockout model. Cas9 mRNA, gRNA targeting the *Ly6a* gene, and a donor vector containing loxP sites were co-injected into fertilized mouse eggs to generate conditional knockout offspring.

(**E**) Schematic diagram of the experimental design and timeline for $Vil1^{creER}$; $Ly6a^{fl/fl}$ mice under *F. nucleatum* and PBS treatments.

(**F** and **G**) Immunoblotting showing LY6A knockout efficiency in colon and small intestine tissues.

(**H** and **I**) immunofluorescence showing LY6A knockout efficiency in colon and small intestine tissues.

(**J**) *F. nucleatum* distribution within crypts of *WT*, $Vil1^{creER}$; $Ly6a^{fl/fl}$, and *Ly6a-DTA* mice.

Scale bars: 100 μm.

Supplemental Figure 7. *F. nucleatum* induced high expression of RPS14 in LY6A^{+} stem cell, Related to Figure 4.

(**A**) Representative immunofluorescence co-staining of Ki67 and RPS14 in tumors from PBS- and *F. nucleatum*-treated mice.

(**B**) Design strategy of *Rps14*$^{fl/fl}$ conditional knockout, *Rps14* is located on chromosome 18 of mice. Using CRISPR/Cas9 technology, sgRNA and ssDNA were designed to obtain *Rps14* gene conditional knockout mice through high throughput electro transfer of fertilized eggs.

(**C**) Schematic diagram of the experimental design and timeline for *Ly6a*^{creERT2}*; Rps14*$^{fl/fl}$ mice under *F. nucleatum* and PBS treatments.

(**D**) Genotyping of tail samples from indicated mice 4 days after tamoxifen injection to confirm Cre expression and *Rps14* deletion.

(**E**) Statistics on the number of tumors larger than 200 um in *WT* and *Ly6a*^{creERT2}*; Rps14*$^{fl/fl}$groups with *F. nucleatum* treatment.

(**F**) In the experimental diagram, *Ly6a*^{creERT2}*; H11*$^{G/R}$ mice mated with *Ly6a*^{creERT2}*; Rps14*$^{fl/fl}$ mice, and the resulting offspring mice were injected with tamoxifen, and then LY6A-tdtomato^{+} cells were sorted to generate organoids.

(**G**) Percentage of LY6A-tdTomato^{+} cells in epithelia from *Ly6a*^{creERT2}*; H11*$^{G/R}$*; Rps14*$^{fl/fl}$ mice.

(**H**) Light microscopy showing tdTomato fluorescence intensity in organoids.

(**I**) Morphology of *Ly6a*^{creERT2}*; H11*$^{G/R}$*; Rps14*$^{fl/fl}$ organoids treated with *F. nucleatum* compared to PBS.

(**J**) Relative growth of *Ly6a*^{creERT2}*; H11*$^{G/R}$*; Rps14*$^{fl/fl}$ organoids treated with PBS or *F. nucleatum*.
Scale bars: 100 μm.
Statistics: (E) and (J): Student's t test (two-tailed).

Supplemental Figure 8. Protein expression and biological function enrichment of mice after *F. nucleatum* intervention were detected by proteome, Related to Figure 4.

(**A**) Heatmap showing changes in protein expression profiles in tumor tissues treated with PBS or *F. nucleatum* in *WT*, *Vil1l*^{creER2}*; Ly6a*$^{fl/fl}$, and *Ly6a*^{creERT2}*; Rps14*$^{fl/fl}$ mice.

(**B**) KEGG pathway enrichment analysis of differential proteins in tumor tissues treated with *F. nucleatum* compared to PBS in *WT* mice.

(**C**) KEGG pathway enrichment analysis of differential proteins in tumor tissues treated with *F. nucleatum* compared to PBS in *Vil1*^{creERT2}*; Ly6a*$^{fl/fl}$ mice.

(**D**) KEGG pathway enrichment analysis of differential proteins in tumor tissues treated with *F. nucleatum* compared to PBS in *Ly6a*^{creER2}*; Rps14*$^{fl/fl}$ mice.

Supplemental Figure 9. scRNA-seq revealed the RPS14 expression in RSCs of human patients with *F. nucleatum* infection, Related to Figure 5.

(**A**) Two-dimensional UMAP plot of single-cell RNA sequencing data (n = 6 para-cancerous tissues, n = 6 tumor tissues).

(**B**) Two-dimensional UMAP plot with color-coded cells labeled according to identity (normal controls: red, tumor: blue).

(**C**) Proportion of RSCs in para-cancerous and cancerous tissues.

(**D**) Feature plot showing the location of RSC markers.

(**E**) FISH assay detecting *F. nucleatum* distribution (20×, Cy5-conjugated, purple) in para-cancerous and cancerous tissues in humans.

Scale bars: 100 μm.

Supplemental Information

Supplemental Table 1. The *F. nucleatum* proteins was identified as a potential interactor with LY6A by mass spectrometry, Related to Figures 3

Supplemental Table 2. Synopsis of pathological data within a clinical cohorts, Related to Figures 1,5

Supplemental Table 3. Enumeration of gene expression profiles in each subtype through single-cell sequencing analysis, Related to Figures 1,5
