# Supplementary material for: Fusobacterium nucleatum promotes colorectal cancer through neogenesis of tumor stem cells
Source: J Clin Invest. 2025 Feb 3;135(3):e181595. doi: 10.1172/JCI181595 (PMC11785920; doi:10.1172/JCI181595)

Full unedited blot/gel for Figure 3B

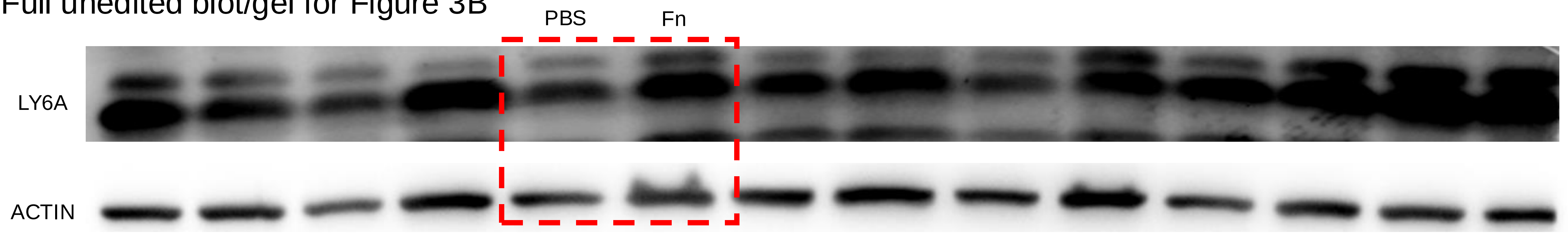

Full unedited blot/gel for Figure 3C

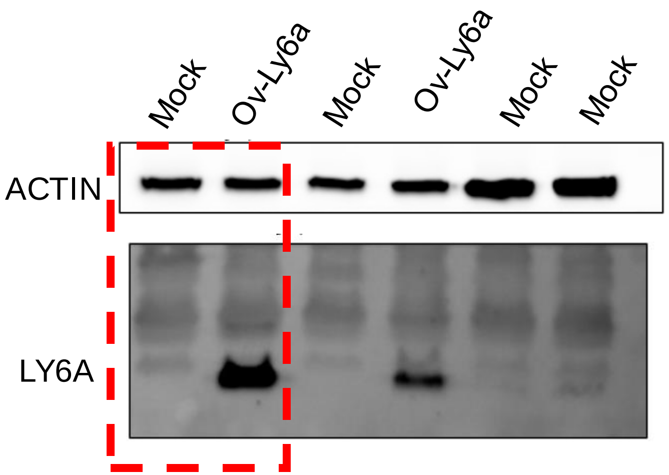

Full unedited blot/gel for Figure 3E

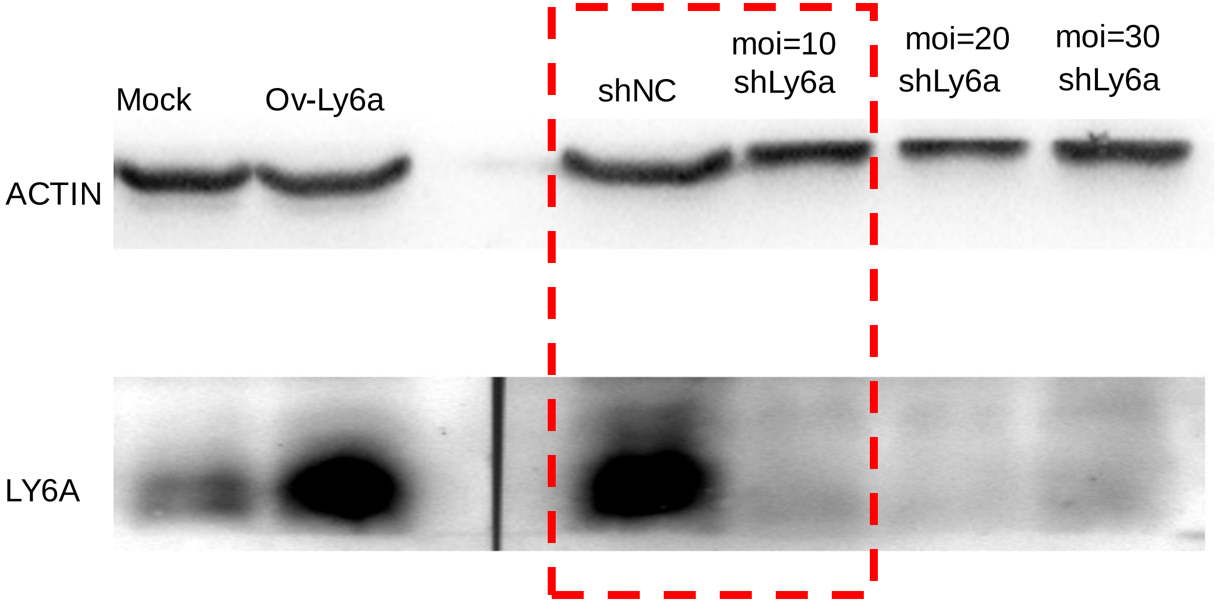

Full unedited blot/gel for Figure 3H

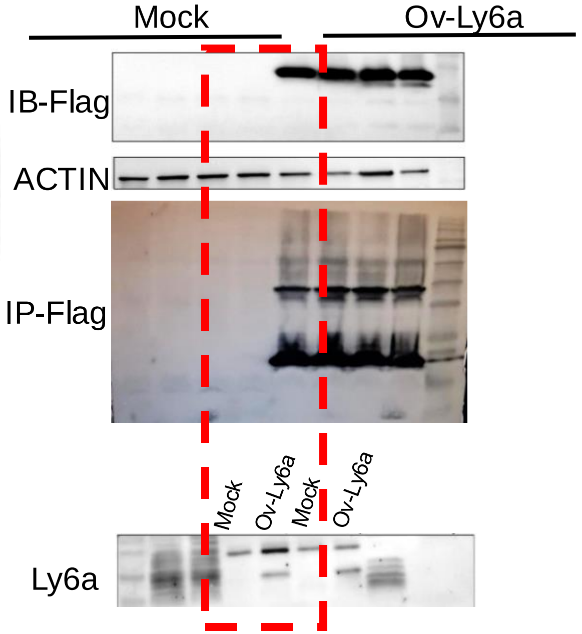

Full unedited blot/gel for Figure 4D

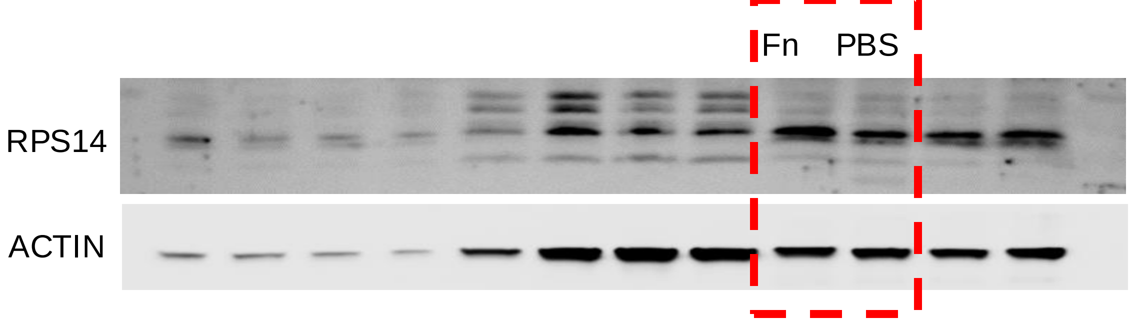

Full unedited blot/gel for Figure 4H

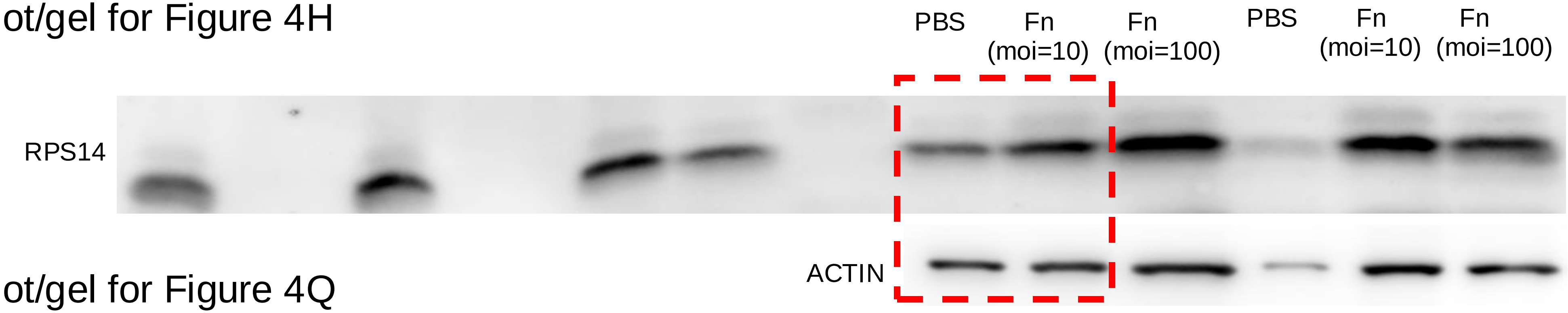

Full unedited blot/gel for Figure 4Q

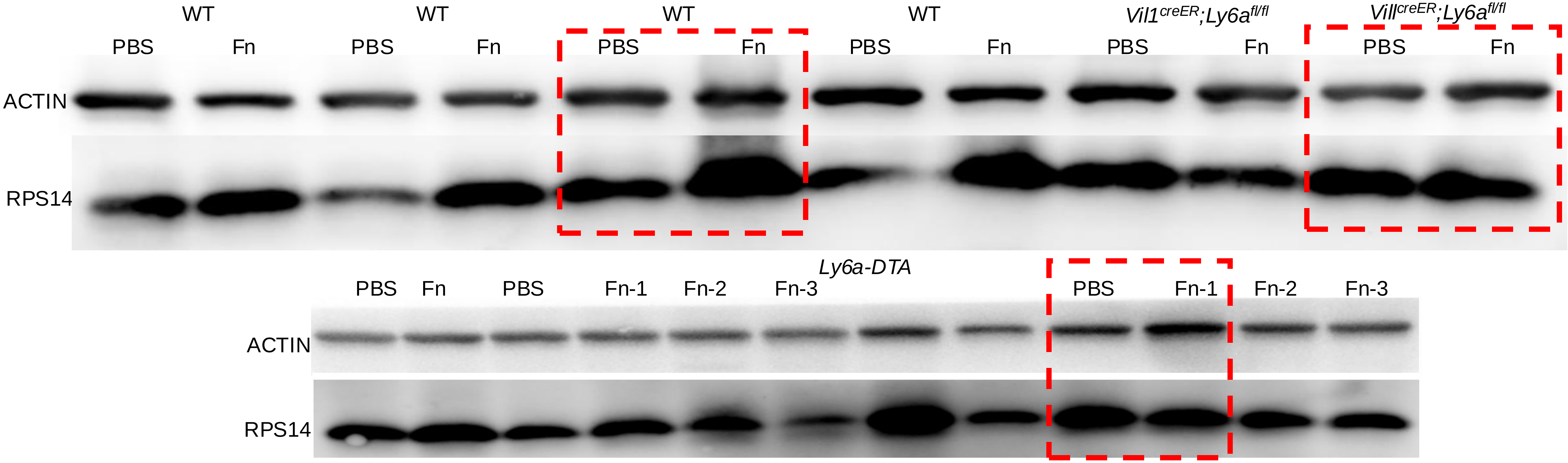

Full unedited blot/gel for Supplemental Figure 5D

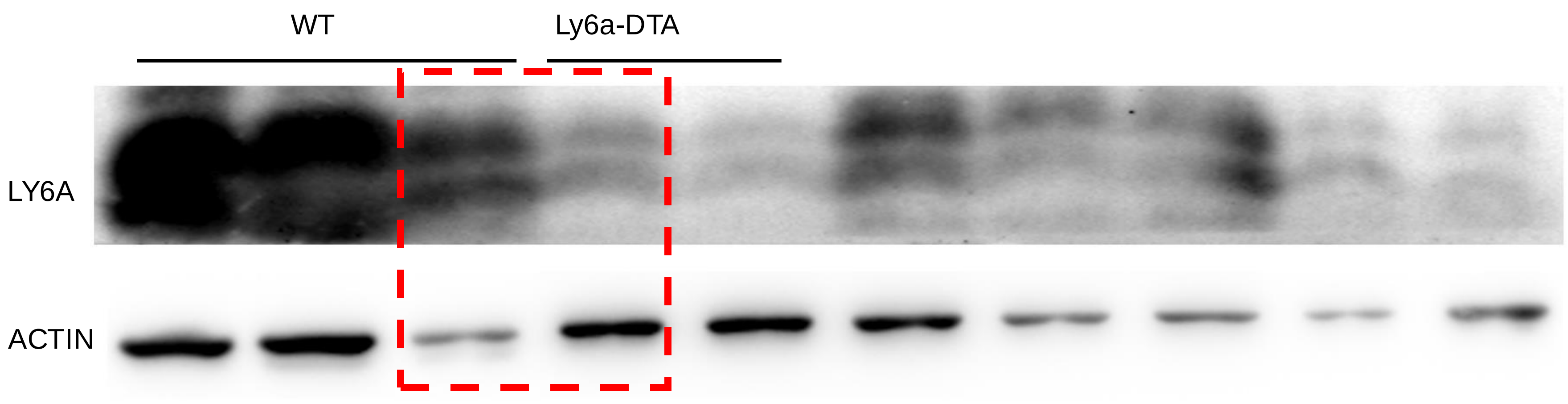

Full unedited blot/gel for Supplemental Figure 6, F and G

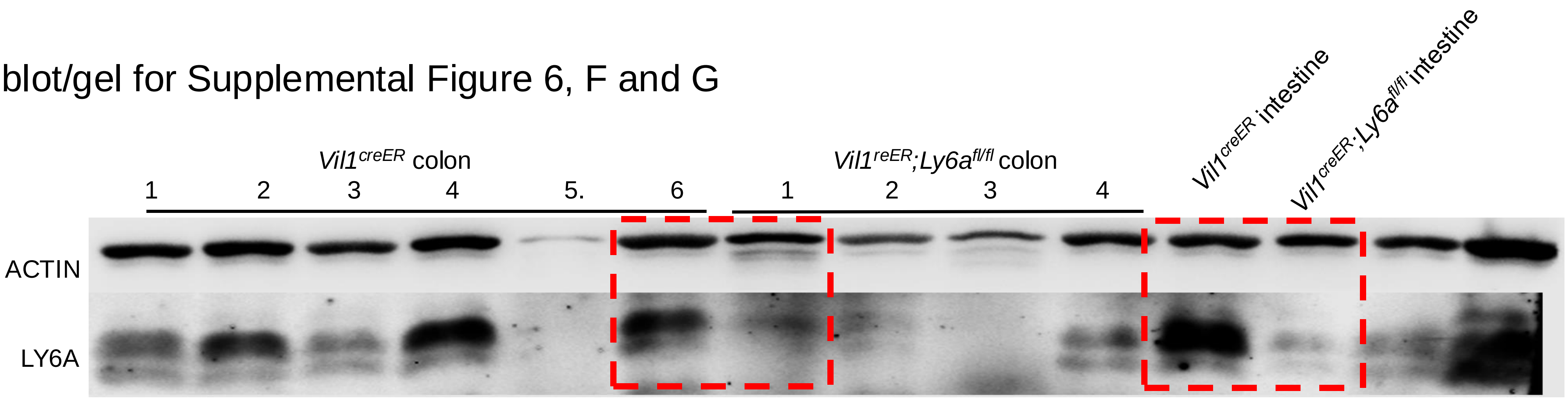

Supplement: Unedited blot and gel images [file jci-135-181595-s185.pdf]
